# Trifluoromethoxy- and Fluorobenzhydryl-Tuned Nickel Catalysts for Polyethylene Elastomers

**DOI:** 10.3390/molecules30132706

**Published:** 2025-06-23

**Authors:** Ming Liu, Min Sun, Yanping Ma, Yizhou Wang, Mingfeng Li, Wen-Hua Sun

**Affiliations:** 1 State Key Laboratory of Petroleum Molecular & Process Engineering, SINOPEC Research Institute of Petroleum Processing Co., Ltd., Beijing 100083, China; liuming.ripp@sinopec.com (M.L.); sunmin.ripp@sinopec.com (M.S.); 2 Key Laboratory of Engineering Plastics, Beijing National Laboratory for Molecular Sciences, Institute of Chemistry Chinese Academy of Sciences, Beijing 100190, China; wangyizhou13@iccas.ac.cn

**Keywords:** nickel catalysts, dual steric-electronic tuning, highly branched polyethylenes, high-temperature polymerization, thermoplastic elastomers (TPEs)

## Abstract

A series of *para*-trifluoromethoxy-substituted and fluorobenzhydryl-functionalized 1,2-bis(imine)acenaphthene ligands: 1-[2,6-{(4-F-C_6_H_4_)_2_CH}_2_-4-F_3_COC_6_H_2_N]-2-(ArN)C_2_C_10_H_6_ (Ar = 2,6-Me_2_C_6_H_3_ **L1**, 2,6-Et_2_C_6_H_3_ **L2**, 2,6-iPr_2_C_6_H_3_ **L3**, 2,4,6-Me_3_C_6_H_2_ **L4**, 2,6-Et_2_-4-MeC_6_H_2_ **L5**), were synthesized and used to generate their corresponding nickel(II) bromide complexes (**Ni1**–**Ni5**). Elemental analysis, ^19^F NMR, and FT-IR spectroscopy were employed to characterize these five nickel complexes. Single-crystal X-ray diffraction of **Ni2** and **Ni4** confirmed distorted tetrahedral geometries. Upon activation with either EtAlCl_2_ (ethylaluminum dichloride) or EASC (ethyl aluminum sesquichloride), these complexes showed exceptional high activities (up to 22.0 × 10^6^ g PE mol^−1^ (Ni) h^−1^) and remarkable thermal stability (4.82 × 10^6^ g PE mol^−1^(Ni) h^−1^ at 80 °C) towards ethylene polymerization. The resulting polyethylenes are highly branched, with the type and extent of branches tunable by temperature, solvent, and co-catalyst choice. Moreover, these polymers demonstrated excellent tensile strength (*σ_b_* up to 20.7 MPa) and elastic recovery (up to 58%), characteristic of thermoplastic elastomers (TPEs). These results highlight the dual role of trifluoromethoxy and fluorobenzhydryl groups in enhancing catalytic performance and polymer properties.

## 1. Introduction

Thermoplastic olefin elastomers (TPEs) have emerged as indispensable polymeric materials due to their excellent mechanical properties, weather resistance, and processability. Their inherent compatibility with polyolefins and cost-effectiveness in enhancing low-temperature toughness have driven widespread applications across automotive, construction, footwear, and consumer goods [1,2,3]. Conventionally synthesized through ethylene/α-olefin copolymerization (e.g., 1-butene, 1-hexene), TPE production relies on advanced catalytic technologies such as Dow Chemical (Midland, MI, USA) Insite™ solution polymerization [4], ExxonMobil’s Exxpol [5,6], and SABIC SK (Ulsan, South Korea) Nexlene™ processes [5]. However, the pursuit of more efficient catalytic systems capable of precise polymer architecture control remains a critical challenge in polyolefin chemistry.

A pivotal breakthrough occurred in the 1990s when Brookhart et al. discovered that α-diimine Ni/Pd complexes could serve as single-center catalysts for the homopolymerization of ethylene, generating branched polyethylene (PE) [7,8] via a chain-walking mechanism [9,10,11,12]. While this innovation sparked intensive research into late-transition metal catalysts [13,14,15,16,17,18,19,20,21,22,23,24], practical implementation was hindered by inherent limitations: insufficient thermal stability and low activity at industrially relevant temperatures. Recent advancements in iron-based α-olefin polymerization catalysts [25] have revitalized interest in addressing these challenges through innovative ligand design.

**Chart 1:**
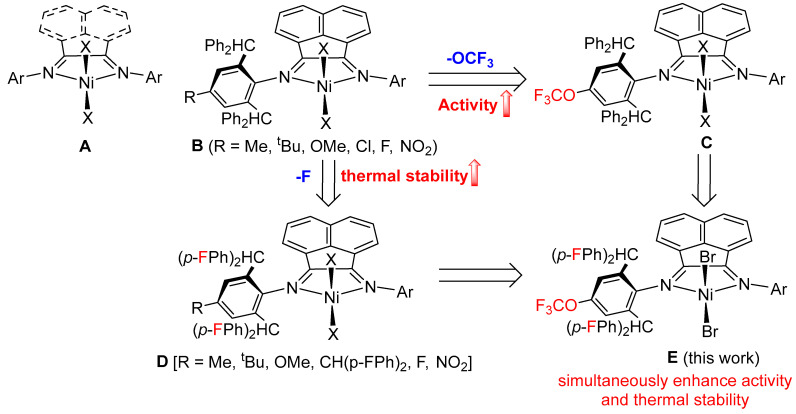
Structural evolution of *N*,*N*′-diiminoacenaphthene-nickel(II) halide precatalysts (**A**–**E**).

The catalytic performance of α-diimine nickel (II) complexes (**A**, Chart 1) is profoundly governed by structural modifications to the ligand framework, particularly through adjustments to the backbone geometry and *N*-aryl substituents. Strategic incorporation of bulky groups (such as benzhydryl and its derivatives, or cycloalkyl groups) [26,27,28,29,30,31,32] and para-substituents possessing varied electronic characteristics (-Me, -*t*Bu, -OMe, -Cl, -F, -NO_2_) enables precise modulation of both steric protection and electronic environment at the metal center. Bulky substituents can effectively improve the thermal stability of catalysts by suppressing deactivation pathways at higher temperature [28,29,33,34], while these *para*-substituents influence the activities of catalysts by influencing the electron density of the metal center.

For instance, our group demonstrated that 2,6-benzhydryl-substituted asymmetric α-diimine-nickel(II) complexes (**B**, Chart 1) generate high-molecular-weight, branched PE with activities correlating to the substituent electronic effects (Me, *t*-Bu, OMe, Cl, F, NO_2_) [35,36,37,38] at the *para*-positions of the *N*-aryl. Notably, catalysts bearing electron-withdrawing substituents tend to exhibit higher activity. When we introduced the strongly electron-withdrawing -OCF_3_ group into the catalyst framework (**C**, Chart 1), the net positive charge at the nickel center was increased, boosting its activity up to 15.1 × 10^6^ g PE mol^−1^ (Ni) h^−1^. Remarkably, replacing conventional toluene with n-hexane as the reaction solvent further enhanced both catalytic performance and thermal stability [39].

Fluorine substitution has emerged as a powerful strategy for improving catalytic performance. Incorporating distal fluorine atoms into benzhydryl group (**D**, Chart 1) (Me [40], *t*-Bu [41], OMe [42], CH(*p*-FPh_2_) [43], F [44], NO_2_ [45]) markedly improved the thermal stability relative to non-fluorinated analogs (**B**, Chart 1). For example, **D_NO2_**, containing strong electron-withdrawing nitro and fluorine substituents, exhibited superior catalytic activity and thermal stability compared to **B_NO2_** complex [38,45]. The **D*_t_*_Bu_**/EASC catalytic system maintained high catalytic activity at 90 °C, and the resulting highly branched PE exhibited typical thermoplastic elastomer (TPE) characteristics [41].

Building on these insights, to synergistically boost both catalytic activity and thermal stability, we devised a dual-functional modification strategy herein: simultaneous installation of a strongly electron-withdrawing trifluoromethoxy group at the *N*-aryl *para*-position and distal fluorine atoms into the benzhydryl groups (**E**, Chart 1). The impact of these modifications on ethylene polymerization were systematically explored by exploring variables such as the ligand structure, co-catalyst choice, solvent (toluene vs. n-hexane), polymerization temperature, running time, and ethylene pressure. Exceptional performance was disclosed at industrially relevant temperatures (up to 80 °C), producing highly branched PE with tunable architectures. Comprehensive characterization (^1^H/^13^C/^19^F NMR, FT-IR, GPC, DSC, XRD, etc.) was used to confirm structures of the ligands, complexes, and PE.

## 2. Results and Discussion

### 2.1. Synthesis and Characterization of the Ligands and Complexes

**Scheme 1:**
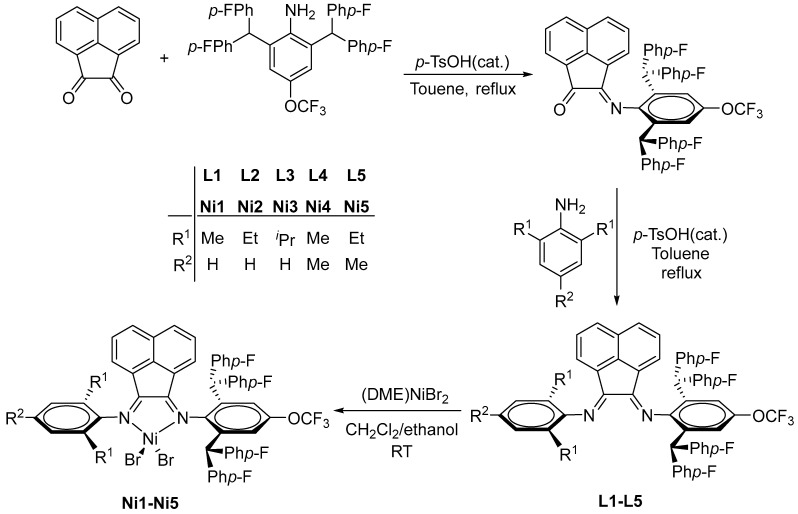
Synthesis of **L1**–**L5** and their corresponding nickel(II) bromide complexes **Ni1**–**Ni5**.

The *N*,*N*′-diimineacenaphthenes **L1**–**L5** 1-[2,6-{(4-FC_6_H_4_)_2_CH}_2_-4-F_3_COC_6_H_2_N]-2- (ArN)C_2_C_10_H_6_ (Ar = 2,6-Me_2_C_6_H_3_ (**L1**), 2,6-Et_2_C_6_H_3_ (**L2**), 2,6-iPr_2_C_6_H_3_ (**L3**), 2,4,6-Me_3_C_6_H_2_ (**L4**), 2,6-Et_2_-4-MeC_6_H_2_ (**L5**)), were prepared via a two-step process (Scheme 1).

Firstly, 2-[2,6-bis(4,4′-difluorobenzhydryl)-4-(trifluoromethoxy)phenylimino]-acenaphthylen-1-one, was synthesized in reasonable yield by the acid catalyzed condensation reaction of acenaphthylene-1,2-dione with one molar equivalent of 2,6-bis(4,4′-difluorobenzhydryl)-4-(trifluoromethoxy)aniline [40,41,42,43,44,45]. This imine-ketone then reacted with the corresponding aniline to afford **L1**–**L5** in moderate yield (22.5–38.0%) (Scheme 1). All ligands were characterized by ^1^H/^13^C/^19^F NMR (Figures S1–S15), FT-IR spectroscopy, and elemental analysis, confirming their structural integrity. Nickel(II) bromide complexes **Ni1**–**Ni5** were generated in good yields (85–92%) by treatment of **L1**–**L5** with (DME)NiBr_2_ (DME = 1,2-dimethoxyethane) in a mixed solvent system composed of dichloromethane and ethanol at room temperature (Scheme 1). All five complexes were characterized by elemental analysis, FT-IR and ^1^H/^19^F NMR spectroscopy (Figures S16–S25). Moreover, the molecular structures of **Ni2** and **Ni4** were confirmed by single-crystal X-ray diffraction analysis (see Table S1: Crystal data and structure refinement for **Ni2** and **Ni4**).

**Figure 1:**
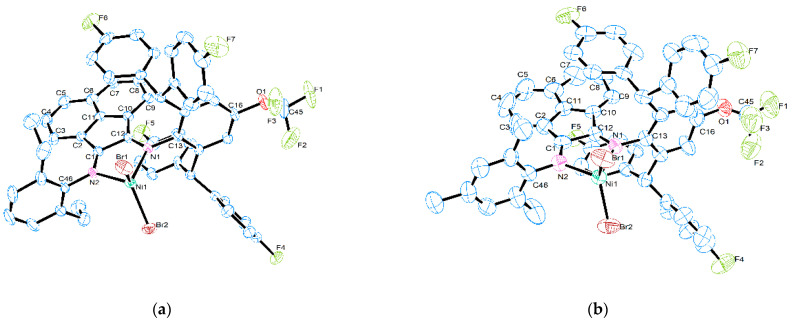
ORTEP diagram of **Ni2** (**a**) and **Ni4** (**b**) with the thermal ellipsoids shown at the 50% probability level. All hydrogen atoms have been omitted for clarity.

Single crystals of **Ni2** and **Ni4** of suitable quality for the X-ray determinations were grown by layering diethyl ether into their dichloromethane solutions at ambient temperature. The molecular structures of **Ni2** and **Ni4** (Figure 1) revealed distorted tetrahedral geometries with the nickel center coordinated by two bromide ligands and the two nitrogen donors from the *N*,*N*′-chelating bis(imine)acenaphthene, as is similar to those reported previously [40,44].

Key bond parameters including bond lengths and angles were listed in Table 1. The N1-Ni1-N2 bite angle of 82.73(12)° in **Ni2** is close to 82.6(3)° in **Ni4**, as is the same for Br1-Ni-Br2 bite angles (126.05(3)° (**Ni2**) vs. 126.56(7)° (**Ni4**)). Nickel-nitrogen bond lengths of Ni1–N1 [ 2.037(3) Å (**Ni2**), 2.041(7) Å (**Ni4**)] are slightly longer than those of Ni1-N2 [2.022(3) Å (**Ni2**), 2.011(7) Å (**Ni4**)], reflecting subtle steric variations between the aryl substituents. The C=N_imine_ bond lengths for both complexes range from 1.261(11) to 1.290(4) Å, which are typical for this functional group [24]. Each imine unit is positioned nearly co-planar with the adjacent acenaphthene unit. The plane of the *N*-aryl group linked to N1 is almost perpendicular to the acenaphthene plane, with dihedral angles of 89.37° for **Ni2** and 89.04° for **Ni4**. On the other hand, due to the smaller steric hindrance, axial rotation is easier for the second *N*-aryl group (aryl = 2,6-Et_2_C_6_H_3_ **Ni2**; 2,4,6-Me_3_C_6_H_2_ **Ni4**), showing some variation [87.09° for **Ni2**, 87.41° for **Ni4**]. Notably, the F_3_C–O bond has double bond character with distance values of 1.342(6) Å for **Ni2**, 1.23(5) Å for **Ni4**, suggesting partial ionic resonance contribution from the limiting form ArO^+^=CF^2^F^−^ [46]. Meanwhile, there are no significant intermolecular contacts.

^19^F NMR spectra of **L1**–**L5** (Figure S11–S15) and **Ni1**–**Ni5** (Figure S21–S25) corroborate ligand coordination. Taking **Ni1** as an example, the spectra were compared with that of the free ligand **L1**. For **L1**, two distinct signals at δ −116.12 and −116.35 arise from restricted rotation of the ortho-aryl groups leading to inequivalent CH(4-FC_6_H_4_)_a_(4-FC_6_H_4_)_b_ groups. Upon complexation (**Ni1**), these signals split further (δ −115.73 and −116.07), consistent with previous studies [41]. The para-trifluoromethoxy resonance shifts upfield slightly from δ −58 in **L1** to δ −57 in **Ni1**, reflecting electronic modulation at the nickel center [39]. FT-IR spectra confirm effective coordination of the two imine nitrogen atoms to the nickel center via redshifted ν_C = N_ absorptions (ligands: 1672–1635 cm^−1^; complexes: 1649–1618 cm^−1^).

### 2.2. Ethylene Polymerization

#### 2.2.1. Co-Catalyst Screening with **Ni1** for Ethylene Polymerization

The nickel-based catalyst precursor **Ni1** was evaluated in ethylene polymerization using five alkylaluminum reagents co-catalysts: methylaluminoxane (MAO), modified methylaluminoxane (MMAO), ethylaluminum dichloride (EtAlCl_2_), ethylaluminum sesquichloride (EASC), and diethylaluminum chloride (Et_2_AlCl)]. Polymerizations were conducted in toluene with ethylene pressure of 10 atm at 30 °C for 30 min, with the results summarized in Table 2.

All alkylaluminum-activated **Ni1** catalysts exhibited high ethylene polymerization activity, producing PEs with high molecular weight (~10^5^ g mol^−1^) and low melting temperatures (T_m_ < 100 °C) indicative of a branched polymer microstructure. The molecular weight distributions were unimodal in the range of 2.48–4.03, indicating single site active species. With regard to the three chloroalkylaluminums, EtAlCl_2_ exhibits the highest activity, presumably attributed to its stronger Lewis acidity, enabling more efficient activation of the metal center [47]. This phenomenon is contrasts to previously reported findings [48]. Among these co-catalysts screened, EtAlCl_2_ and EASC exhibited the highest catalytic activities, reaching 13.2 × 10^6^ g PE mol^−1^ (Ni) h^−1^ and 8.78 × 10^6^ g PE mol^−1^ (Ni) h^−1^, respectively. These values represent an enhancement compared to MAO and MMAO under identical conditions. Due to their superior performance, EtAlCl_2_ and EASC were selected for subsequent optimization of polymerization conditions (such as Al/Ni ratio, polymerization temperature, reaction time, and ethylene pressure) and evaluation with other catalyst precursors.

#### 2.2.2. Optimizing Ethylene Polymerization Parameters Using **Ni1**–**Ni5**/EtAlCl_2_

In this section, we systematically investigated the effects of Al:Ni molar ratio, polymerization temperature, reaction time, ethylene pressure, and ligand structure on ethylene polymerization using **Ni1**–**Ni5** precatalysts activated by EtAlCl_2_ (Table 3).

First, we systematically evaluated the polymerization behavior across reaction temperatures ranging from 30 °C to 80 °C, under fixed conditions: 10 atm ethylene pressure, Al/Ni = 300, 30 min (runs 1–6, Table 3). The results revealed that catalytic activity reached its peak at 40 °C (14.2 × 10^6^ g PE mol^−1^ (Ni) h^−1^), followed by a decrease at higher temperatures attributed to partial deactivation of active species and reduced ethylene solubility [49,50]. Despite this, the activity value of 2.76 × 10^6^ g PE mol^−1^ (Ni) h^−1^ at 80 °C remained notably high, underscoring the positive influence of bulky substituents and the distal F group on thermal stability [39,40,41]. Concurrently, the polymer molecular weight gradually decreased from 5.96 × 10^5^ g mol^−1^ to 2.26 × 10^5^ g mol^−1^ with increasing the temperature from 30 to 80 °C, due to accelerated chain transfer rate [45,51,52,53]. Notably, over this temperature span, all polymers displayed narrow dispersity (M_w_/M_n_ range: 1.73–3.55) and a unimodal distribution (Figure S26), confirming a single active species participated in the polymerization process. The polymer’s melting point (T_m_) inversely correlated with the reaction temperature, likely due to reduced molecular weight and increased branching density at elevated temperatures, leading to reduced crystallinity. These results suggest that the high temperatures promote chain walking isomerization, enhancing branching [54].

Next, we fixed the polymerization temperature at 40°C and varied the Al:Ni molar ratio from 100:1 to 300:1 to assess the influence of co-catalyst concentration (runs 2, 7–10, Table 3). As the Al:Ni molar ratio increased, catalytic activity initially rose, reaching a peak of 17.2 × 10^6^ g PE mol^−1^ (Ni) h^−1^ at 200:1. Beyond this optical ratio, the activity declined, indicating that excess EtAlCl_2_ concentration may facilitate chain transfer from the Ni center to the Al center, resulting in chain termination [55].

Further analysis of reaction time dependence (runs 2, 11–14, Table 3) revealed a distinct inverse relationship between time and activity. The highest activity was achieved within the first 5 min (33.2 × 10^6^ g PE mol^−1^ (Ni) h^−1^), followed by a gradual decline, suggesting rapid initial activation of the Ni center by EtAlCl_2_, and progressive deactivation of active species over time [41]. Nonetheless, catalytic activity remained robust at 60 min [11.8 × 10^6^ g PE mol^−1^ (Ni) h^−1^], highlighting the stability of the residual active species and their prolonged catalytic lifetime. Meanwhile, the PE molecular weight increased steadily from 2.78 × 10^5^ g mol^−1^ to 5.08 × 10^5^ g mol^−1^, indicating that although some active sites deactivated, others retained activity, enabling continued chain growth over time. When polymerization pressures were lowered (10 atm, 5 atm, 1 atm), both catalytic activity and polymer molecular weight were found to diminish, likely due to the reduced ethylene concentration at lower pressure [38,44].

Under optimized conditions (Al/Ni = 200, 40 °C, 30 min, 10 atm), ligand modifications in **Ni1**–**Ni5** significantly impacted catalytic performance. All complexes exhibited high ethylene polymerization catalytic activity [6.74–17.2 × 10^6^ g PE mol^−1^ (Ni) h^−1^], producing high molecular weight PE (M_w_: 3.57–6.68 × 10^5^ g mol^−1^) with low melting points (*T*_m_: 51.1–63.8 °C), indicating a high degree of branching. The catalytic activity followed the order: **Ni1** > **Ni4** > **Ni5** > **Ni2** > **Ni3**, indicating that catalytic systems with smaller substituents demonstrate superior activity, by minimizing steric hindrance to ethylene coordination and insertion at the active site [45]. Conversely, **Ni3** bearing bulkier substituents, exhibited the lowest activity by yield the PE with highest molecular weight (6.68 × 10^5^ g mol^−1^, run 18, Table 3), suggesting that bulky substituents protect active species and mitigate chain termination.

#### 2.2.3. Optimizing Ethylene Polymerization Parameters Using **Ni1**–**Ni5**/EASC

Similarly, we evaluated ethylene polymerization performance of **Ni1**–**Ni5** complexes using EASC as the co-catalyst, with experimental results listed in Table 4. The **Ni1**–**Ni5**/EASC system also exhibited efficient catalytic activity, yielding high molecular weight PE with narrow distribution and low melting points, indicative of branched polymer microstructures.

**Figure 2:**
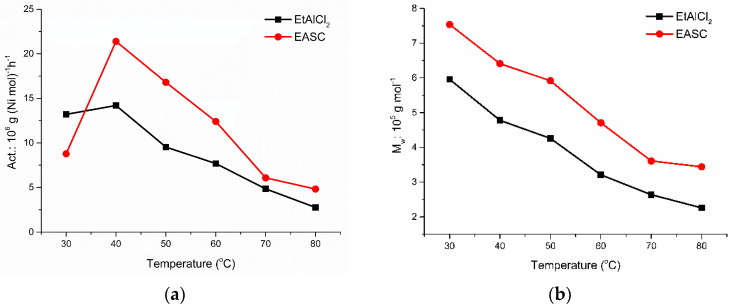
Temperature-dependent (**a**) activity and (**b**) molecular weight trends using **Ni1**/EASC vs. **Ni1**/EtAlCl_2_.

As the temperature rises from 30 °C to 80 °C (run 1–6, Table 4), the catalytic activity of **Ni1**/EASC initially increases to a maximum of 21.4 × 10^6^ g PE mol^−1^(Ni) h^−1^ at 40 °C, followed by a gradual decline, while polymer molecular weight steadily decreased—a pattern mirroring the **Ni1**/EtAlCl_2_ system (Figure 2). Notably, **Ni1**/EASC showed 1.5-fold higher peak activity than **Ni1**/EtAlCl_2_ [21.4 vs. 14.2 × 10^6^ g (PE)·mol^−1^(Ni)·h^−1^] and retained superior thermal stability at elevated temperatures. At 80 °C, **Ni1**/EASC maintained 75% higher activity [4.82 × 10^6^ vs. 2.76 × 10^6^ g (PE)·mol^−1^(Ni)·h^−1^], underscoring the beneficial influence of EASC as co-catalyst on the thermal stability of the catalytic system. Moreover, due to enhanced chain transfer rate at higher temperatures, the molecular weight of PEs falls as temperature increases: At 80 °C, M_w_ drops by 54% for **Ni1**/EASC and by 63% for **Ni1**/EtAlCl_2_ compared with their values at 30 °C (Figure 2b).

Next, we set the reaction temperature to 40 °C and varied the Al:Ni molar ratio from 250:1 to 450:1 to examine the impact of co-catalyst concentration on polymerization performance (runs 2, 7–10, Table 4). The activity peaked at 350:1 [22.0 × 10^6^ g PE mol^−1^ (Ni) h^−1^], higher than the optical ratio for EtAlCl_2_ (200:1), attributed to the distinct properties of the two alkylaluminum reagents in EASC. Notably, this activity value surpasses most reported for *N*,*N*′-nickel catalysts under similar reaction conditions [24,35−45], except for one cycloalkyl substituted analog [22]. At an Al/Ni ratio of 450:1, the PE’s relative molecular weight reached its maximum value of 7.16 × 10^5^ g mol^−1^.

**Figure 3:**
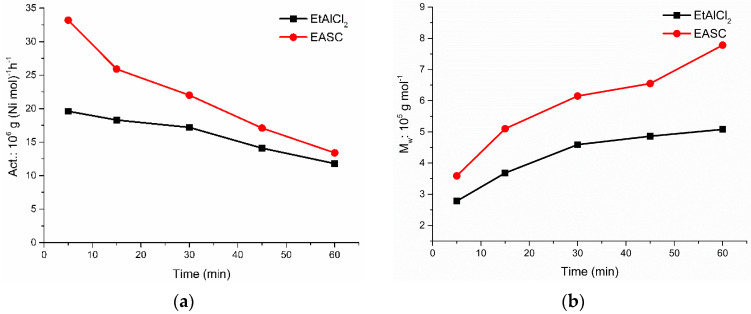
Time-dependent (**a**) activity decay and (**b**) molecular weight evolution using **Ni1**/EASC vs. **Ni1**/EtAlCl_2_.

Under optimal conditions, **Ni1**/EASC exhibits significantly faster initial activation (33.2 × 10^6^ vs. 19.6 × 10^6^ g PE mol^−1^(Ni) h^−1^ 5 min) but accelerated deactivation (60% activity loss at 60 min) compared to **Ni1**/EtAlCl_2_ (40% loss) (Figure 3a). Despite this, **Ni1**/EASC consistently yielded PE with higher molecular weight (runs 8, 11–14, Table 4) than **Ni1**/EtAlCl_2_ (e.g., 7.78 × 10^5^ vs. 5.08 × 10^5^ g mol^−1^ at 60 min; Figure 3b), underscoring its superior propagation efficiency under similar conditions. Likewise, reducing ethylene pressure diminishes both activity and polymer molecular weight in the **Ni1**/EASC system (runs 8, 15–16, Table 4), mirroring trends observed for **Ni1**/EtAlCl_2_.

Having identified the optimal reaction conditions for **Ni1**/EASC (Al:Ni = 350:1, 30 °C, 30 min, 10 atm ethylene), we further investigated the influence of ligand structural variations on polymerization performance (runs 2, 17–20, Table 4). The activity of the nickel complexes decreased in the following order: **Ni1** > **Ni5** > **Ni2** ≈ **Ni4** > **Ni3**. While the reasons behind these activity variations remain unclear, it is possible that different counteranions generated during catalyst activation affect the solubility of the complexes. Additionally, the electron-donating methyl group at the para-position of the *N*-aryl substituent appears to negatively affect polymer molecular weight, a phenomenon previously reported [45].

Building on prior findings that solvents critically influence polymerization reactions [39], we explored the impact of *n*-hexane versus toluene on **Ni1**/EASC (Table 5).

Using identical optimal reaction conditions (Al:Ni = 350:1, 40 °C, 30 min, 10 atm ethylene), replacing toluene with n-hexane noticeably reduced catalytic activity [hexane: 2.46 × 10^6^ vs. toluene: 22.0 × 10^6^ g PE mol^−1^ (Ni) h^−1^] but increased polymer molecular weight [hexane: 8.24 × 10^5^ vs. toluene: 6.15 × 10^5^ g mol^−1^]. Further optimization the polymerization using hexane as the solvent showed catalytic activity peaked at 13.7 × 10^6^ g PE mol^−1^ (Ni) h^−1^ when Al:Ni = 550:1 at 40 °C, though still lower than the maximum activity observed in toluene. The cause of this behavior remains uncertain, it may be due to solvent-induced changes in catalyst solubility, solvent polarity, ligand electronic effects, and the ethylene coordination environment. Additionally, polymers synthesized in n-hexane exhibited higher melting points (e.g., 116.8 °C in n-hexane vs. 59.5 °C in toluene, Al/Ni = 350, T = 40 °C), suggesting reduced branching (*vide infra*).

### 2.3. Systematic Investigation of Substituent Effects on Catalytic Performance

#### 2.3.1. Role of the *para*-Trifluoromethoxy (-OCF_3_) Group

**Figure 4:**
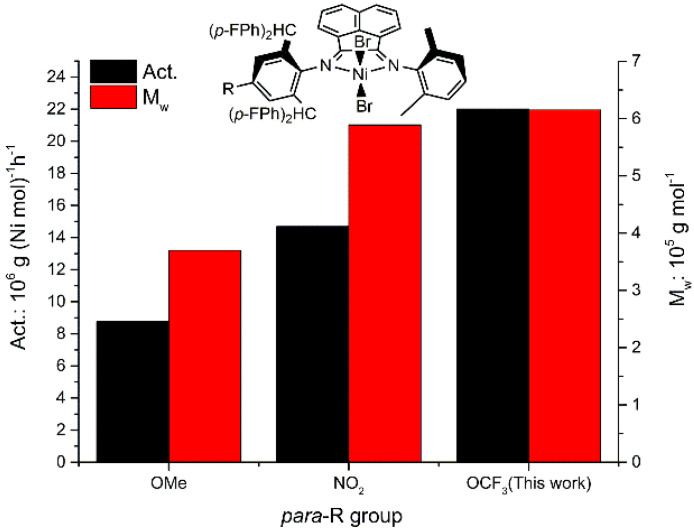
Comparison of activity and M_w_ of the PE generated by **Ni1** (R = OCF_3_) with precatalyst **D** (Chart 1, R = OMe [35], NO_2_ [36]) under identical conditions (EASC, 40 °C, P_C2H4_ = 10 atm, toluene).

To clarify the impact of the *para*-trifluoromethoxy (-OCF₃) group on catalytic activity and molecular weight of the polymer, we compared **Ni1** with two reported asymmetric bis(arylimino)acenaphthene-nickel(II) bromide complexes (**D**, Chart 1) bearing *para*-OMe [42] and *para*-NO_2_ [45] substituents (Figure 4). All polymerization reactions shown in the figure were carried out under identical conditions (EASC co-catalyst, toluene, 30 °C, and 10 atm ethylene).

From Figure 4, it is clear that introducing an -OCF_3_ group at the para-position significantly enhances catalytic activity: **Ni1** achieved 22.0 × 10^6^ g PE mol^−1^ (Ni) h^−1^ at 40 °C, nearly three times that of its para-methoxy (-OMe) analog. This improvement is due to the strong electron-withdrawing nature of the *para*-OCF_3_, which likely polarizes the nickel center, facilitating ethylene coordination and insertion. In contrast, the *para*-nitro (-NO_2_) analog exhibited reduced activity attributed to stronger electron-withdrawing of -NO_2_, which impedes ethylene insertion [45]. Regarding PE molecular weight, all three nickel complexes in Figure 4 produced high molecular weight PEs (10^5^ g mol^−1^), suggesting that the *ortho*-bulky substituents help promote chain growth while effectively suppressing chain-transfer reaction [37]. Among them, the complex with the -OCF_3_ group generated the highest molecular weight PE at 6.15 × 10^5^ g mol^−1^, emphasizing the promoting effect of this strongly electron-withdrawing group on ethylene insertion [45].

#### 2.3.2. Effects of the Distal Fluorine Atom

**Figure 5:**
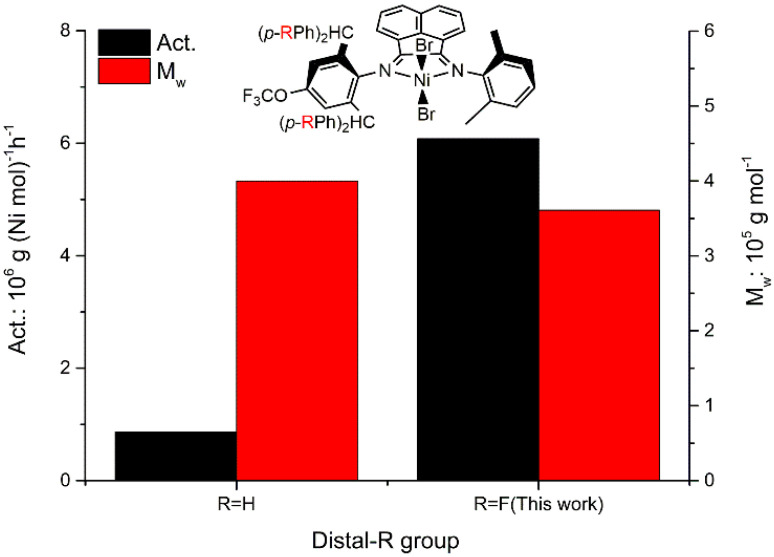
Comparison of activity and M_w_ of the PE generated by **Ni1** (R = F) with precatalyst **C** (Chart 1, R = H [43]) under identical conditions (EASC, 70 °C, Al/Ni = 300, P_C2H4_ = 10 atm, toluene).

To isolate the influence of the distal fluorine substituent, we compared **Ni1** with its non-fluorinated analog **C** [39] (Chart 1) under identical conditions (Figure 5). We found that **Ni1** bearing the distal fluorine exhibits markedly enhanced catalytic activity and remains highly efficient for ethylene polymerization at the industrially relevant temperature (70 °C), presumably due to the enhanced Lewis acidity of the active metal species, leading to more efficient ethylene coordination and insertion. This demonstrates the positive role of the fluorine atom in boosting the catalyst’s thermal stability and suggests excellent prospects for industrial application. In terms of polymer molecular weight, introduction of the distal F atom causes a slight decrease in the resulting PE’s molecular weight, which may attribute to participant of the distal F atoms in chain-terminating interactions via weak C–H···F interactions with the growing polymer chain or solvent. This observation is consistent with previous study [56]. However, due to the bulky ortho substituent and the strongly electron-withdrawing *para*-trifluoromethoxy group, the obtained PE still maintains M_w_ on the order of 10^5^ g mol^−1^.

In summary, by employing a dual-modification strategy with introducing trifluoromethoxy group and distal fluorine substituents, we have dramatically enhanced the activity and thermal stability of the pre-catalyst, enabling efficient ethylene polymerization at industrially relevant temperatures to yield high-molecular-weight PEs.

### 2.4. Branching Properties of the PEs

**Figure 6:**
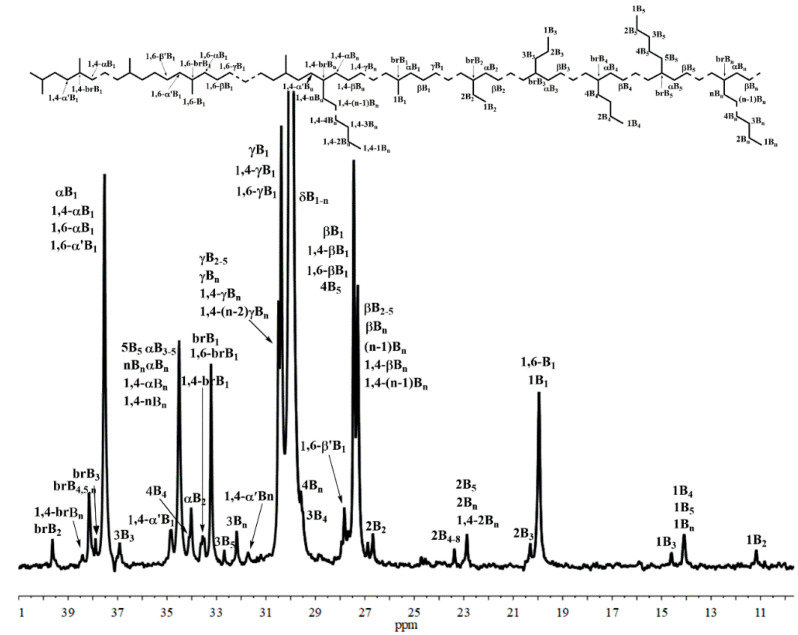
^13^C NMR spectrum of PE-40_EADC/300/T_ along with a segment of the assigned polymer backbone; recorded in C_6_D_4_Cl_2_ at 100 °C.

All PEs synthesized in this study have melting temperatures (T_m_) below 120 °C, characteristic of branched PE (Table 2, Table 3, Table 4 and Table 5). To quantify the effects of temperature, solvent, co-catalyst type and concentration on branching density and topology, ^13^C NMR spectroscopy (measured at 100 °C, using C_6_D_4_Cl_2_ as solvent) was used to analyze selected PE samples (Figure 6 and Figures S27–S31). From these spectra, characteristic signals corresponding to distinct carbon environments in the polymer chain and branches were identified, with results shown in Table 6.

A comparative analysis of PE samples synthesized with EtAlCl_2_ (PE-40_EADC/300/T_, PE-60_EADC/300/T_) and EASC PE-40_EASC/300/T_, and PE-60_EASC/300/T_ revealed that increasing polymerization temperature elevated the degree of branching, while reducing the methyl branch content and promoting other types of branches. This indicates that higher temperatures favor chain walking, which leads to the formation of longer and more complex branched structures [57].

**Table 6:** Branching analysis of selected PE samples *^a^*.

PE Sample *^b^*	Branches/1000 C’s	Methyl Branches/%	Ethyl Branches/%	Propyl Branches/%	Butyl Branches/%	Amyl Branches/%	Longer Branches/%
PE-40_EADC/300/T_	96.5	77.0	5.21	5.50	3.52	2.59	6.17
PE-60_EADC/300/T_	128	70.2	7.29	6.92	5.23	3.04	7.31
PE-40_EASC/300/T_	90.5	80.8	5.63	5.18	3.71	1.07	3.56
PE-60_EASC/300/T_	107	79.4	5.46	5.26	3.26	1.62	5.03
PE-40_EASC/450/T_	102	77.3	5.60	5.75	3.10	3.03	5.21
PE-40_EASC/450/H_	52.6	83.0	4.83	3.70	1.81	1.55	5.10

*^a^* Data determined from the ^13^C NMR spectrum using approaches described by G. B. Galland [58]; *^b^* Naming example: PE-40_EADC/300/T_: 40 °C, EtAlCl_2_, Al:Ni = 300, toluene.

Compared to EtAlCl_2_, PE produced with EASC as a co-catalyst exhibited a lower degree of branching and lower long-chain content. In terms of the co-catalyst concentration, as Al:Ni increased from 300 to 450, the degree of branching in the resulting polymer increased (90.5 to 102 branches/1000 C), which explains why PE-40_EASC/450/T_ has a lower melting point (56.7 vs. 58.7 °C) despite a higher molecular weight (7.16 vs. 6.41 × 10^5^ g mol^−1^; Table 4 run 10 vs. Run 2). Clearly, the choice of solvent affects the degree of branching: under the same conditions, samples obtained in toluene exhibited higher branching (102 branches/1000 C) than those obtained in hexane (52.6 branches/1000 C). This is contrasts to previously reported findings [39], and it seems that the introduction of distal fluorine substituent may perturb solvent-polymer interactions or chain-transfer pathways, though the underlying cause of this phenomenon requires further investigation.

### 2.5. Mechanical Properties of the PE

**Figure 7:**
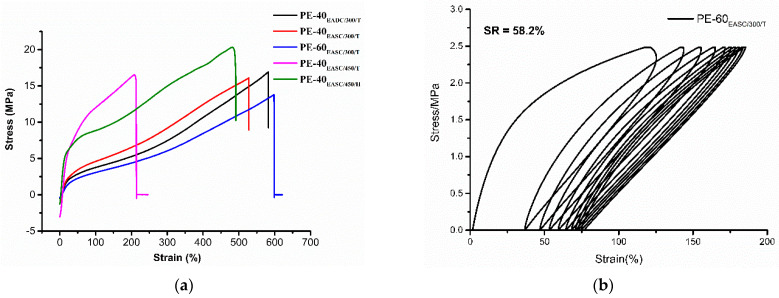
(**a**) Stress–strain curves for PE-40_EADC/300/T_, PE-40_EASC/300/T_, PE-60_EASC/300/T_, PE-40_EASC/450/T_, and PE-40_EASC/450/H_ obtained during monotonic tensile deformation; (**b**) Hysteresis loops for PE-60_EASC/300/T_ obtained using ten cycles at 30 °C.

To explore the mechanical properties of these branched polymers, five samples—PE-_40EADC/300/T_ (run 2, Table 3), PE-40_EASC/300/T_ (run 2, Table 4), PE-60_EASC/300/T_ (run 4, Table 4), PE-40_EASC/450/T_ (run 10, Table 4), and PE-40_EASC/450/H_ (run 2, Table 5) were subjected to monotonic tensile stress–strain testing. Five specimens of each sample were tested, and the average results were calculated to ensure statistical reliability. The tensile test data are shown in Table 7, and the stress–strain curves and hysteresis loops are provided in Figure 7.

We found that these polymers exhibited superior tensile strength compared to those previously reported using nickel-catalysted systems [19,20,35,39,41,50]. In terms of the effects of polymerization conditions, PE-40_EADC/300/T_ (activated with EtAlCl_2_) and PE-40_EASC/300/T_ (activated with EASC) showed comparable tensile strengths, but PE-40_EADC/300/T_, which had a higher degree of branching (96.5 vs. 90.5 branches/1000 C), demonstrated a higher elongation at break (585% vs. 482%). For samples obtained at higher temperatures, crystallinity decreased to 17.0%, resulting in a reduction in tensile strength to 13.7 MPa, while elongation at break increased to 572%. On the other hand, when n-hexane was used as the solvent instead of toluene, PE-40_EASC/450/H_ exhibited improvements in both tensile strength (20.7 MPa) and elongation at break (439%) compared to PE-40_EASC/450/T_. These results indicate that polymer molecular weight, crystallinity, and branching structure significantly influence the tensile properties of PE.

Finally, to assess the elastic recovery performance of the polymers, dynamic mechanical analysis (DMA) was used to perform hysteresis tests on PE-60_EASC/300/T_. The tests were conducted at 30 °C, with each cycle repeated 10 times, as shown in Figure 7b. The elastic recovery rate (SR) was calculated using the standard formula SR = 100(ε_a_ − ε_r_)/ε_a_, where ε_a_ is the applied strain, and ε_r_ is the strain after 10 cycles at zero load. Notably, after 10 cycles, the sample retained good elastic recovery performance with an SR of 58.2%, which can be attributed to the high degree of branching and the relatively high content of long branches in the PE [35]. In summary, these PE samples demonstrated excellent tensile strength, elongation at break, and elastic recovery, making them potential substitutes for commercial thermoplastic elastomers.

## 3. Materials and Methods

### 3.1. Synthesis of 2-(2,6-Bis(bis(4-fluorophenyl)methyl)-4-trifluoromethoxy)acenaphthylen-1-one

To a mixture of 2,6-bis(4,4′-difluorobenzhydryl)-4-(trifluoromethoxy)aniline (4.65 g, 8 mmol), acenaphthylene-1,2-dione (1.60 g, 8.8 mmol), and a catalytic amount of *p*-toluenesulfonic acid (1.25 g), toluene (30 mL) was added. The resulting solution was refluxed for 5.5 h. After the reaction, all volatiles were removed under reduced pressure, and the residue was purified by alumina column chromatography (500/8 petroleum ether/ethyl acetate). The product was obtained as a yellow powder (3.76 g, 63.0%). ^1^H NMR (400 MHz, CDCl_3_, TMS): δ 8.13–8.08 (m, 2H), 7.86 (d, *J* = 8.4 Hz, 1H), 7.78 (t, *J* = 7.8 Hz, 1H), 7.10 (t, *J* = 7.8 Hz, 1H), 6.95 (d, *J* = 6.8 Hz, 9H), 6.81 (s, 2H), 6.79–6.75 (m, 4H), 6.31 (t, *J* = 8.4 Hz, 3H), 5.99 (d, *J* = 6.8 Hz, 1H), 5.40 (s, 2H). ^13^C NMR (100MHz, CDCl_3_, TMS): δ 188.98, 162.98, 162.88, 162.07, 160.44, 159.62, 146.49, 145.81, 142.54, 137.24, 137.12, 136.38, 136.35, 133.82, 132.66, 132.52, 131.27, 131.20, 130.93, 130.86, 130.72, 130.64, 130.56, 130.07, 129.99, 129.53, 129.47, 128.48, 128.14, 127.04, 126.19, 123.34, 122.27, 122.12, 121.53, 120.85, 119.17, 115.88, 115.67, 115.49, 115.28, 115.20, 114.99, 50.51.

### 3.2. Synthesis of Ligands (***L1***–***L5***)

**L1**. To a mixture of 2-(2,6-bis(4,4′-difluorobenzhydryl)-4-trifluoromethoxy) acenaphthylen-1-one (1.49 g, 2.0 mmol), 2,6-dimethylaniline (0.36 g, 3.0 mmol), and a catalytic amount of p-toluenesulfonic acid (0.37 g), toluene (30 mL) was added. The resulting solution was refluxed for 6 h. After the reaction, all volatiles were removed under reduced pressure, and the residue was purified by alumina column chromatography (500/8 petroleum ether/ethyl acetate). The product was obtained as a yellow powder (0.49 g, 27.6%). ^1^H NMR (400 MHz, CDCl_3_, TMS): δ 7.82 (d, *J* = 8.4 Hz, 1H, An-H), 7.75 (d, *J* = 8.0 Hz, 1H, An-H), 7.33 (t, *J* = 7.6 Hz, 1H, An-H), 7.19 (d, *J* = 7.6 Hz, 2H, Ar-H), 7.13–7.09 (m, 1H, Ar-H), 7.06 (t, *J* = 7.6 Hz, 1H, An-H), 7.02–6.94 (m, 8H, Ar-H), 6.87–6.82 (m, 6H, Ar-H), 6.59 (d, *J* = 7.2 Hz, 1H, An-H), 6.32 (t, *J* = 8.4 Hz, 1H, Ar-H), 5.98 (d, *J* = 7.2 Hz, 1H, An-H), 5.59 (s, 2H, -C*H*Ph_2_), 2.19 (s, 6H, -CH_3_). ^13^C NMR (100MHz, CDCl_3_, TMS): δ 164.2, 162.9, 162.0, 161.1, 160.4, 159.6, 148.8, 147.4, 145.4, 139.9, 137.5, 137.5, 136.5, 136.5, 134.2, 131.1, 131.0, 130.7, 130.6, 130.1, 129.3, 129.2, 128.5, 128.3, 128.1, 127.9, 126.7, 124.5, 124.1, 123.6, 122.4, 121.8, 120.9, 119.2, 115.4, 115.2, 115.1, 114.9, 50.9, 50.6, 18.1. ^19^F NMR (470 MHz, CDCl_3_, TMS): δ −58.17, −116.12, −116.35. FT-IR (cm^−1^): 3074 (w), 3043 (w), 2977 (w), 2941 (w), 1662 (m, *v*_C = N_), 1635 (w, *v*_C = N_), 1697 (m), 1506 (s), 1465 (w), 1440 (m), 1257 (w), 1217 (w), 1159 (w), 1095 (w), 1040 (w), 1012 (w), 928 (w), 875 (w), 828 (m), 802 (w), 769 (m). Anal. calcd for C_53_H_35_F_7_N_2_O·H_2_O (848.86): C, 74.99; H, 4.16; N, 3.30. Found: C, 75.24; H, 4.10; N, 3.11%.

**L2**. Using a typical procedure as described for the synthesis of **L1**, **L2** was prepared as a yellow powder (0.51 g, 29.1%). ^1^H NMR (400 MHz, CDCl_3_, TMS): δ 7.80 (d, *J* = 8.0 Hz, 1H), 7.71 (d, *J* = 8.4 Hz, 1H), 7.31 (t, *J* = 7.8 Hz, 1H), 7.25–7.20 (m, 3H), 7.02–6.94 (m, 9H), 6.86–6.83 (m, 6H), 6.58 (d, *J* = 7.2 Hz, 1H), 6.30 (t, *J* = 8.6 Hz, 4H), 5.88 (d, *J* = 7.2 Hz, 1H), 5.60 (s, 2H), 2.70–2.61 (m, 2H), 2.54–2.45 (m, 2H), 1.17 (t, *J* = 7.4 Hz, 6H). ^13^C NMR (100MHz, CDCl_3_, TMS): δ 162.9, 162.0, 161.2, 160.4, 159.6, 147.9, 147.5, 139.9, 137.8, 136.5, 136.4, 134.2, 131.0, 130.7, 130.6, 130.4, 130.00, 129.2, 129.0, 128.3, 127.8, 126.6, 126.4, 124.5, 123.7, 122.9, 121.0, 115.4, 115.2, 115.1, 114, 9, 50.6, 24.5, 14.4. ^19^F NMR (470 MHz, CDCl_3_, TMS): δ −58.18, −116.13, −116.33. FT-IR (cm^−1^): 3053 (w), 2966 (w), 2937 (w), 2878 (w), 1672 (w, *v*_C = N_), 1654 (w, *v*_C = N_), 1598 (w), 1506 (s), 1437 (m), 1253 (w), 1224 (w), 1159 (w), 1095 (w), 1042 (w), 1014 (w), 923 (w), 875 (w), 828 (w), 802(w), 782 (w). Anal. calcd for C_55_H_39_F_7_N_2_O·H_2_O (876.92): C, 75.33; H, 4.48; N, 3.19. Found: C, 75.65; H, 4.32; N, 2.87%.

**L3.** Using a typical procedure as described for the synthesis of **L1**, **L3** was prepared as a yellow powder (0.44 g, 24.1%). ^1^H NMR (400 MHz, CDCl_3_, TMS): δ 7.78 (d, *J* = 8.4 Hz, 1H), 7.68 (d, *J* = 8.4 Hz, 1H), 7.33–7.29 (m, 4H, An-H), 7.02–6.93 (m, 9H), 6.85–6.82 (m, 6H), 6.48 (d, *J* = 7.2 Hz, 1H), 6.27 (t, *J* = 8.6 Hz, 4H), 5.79 (d, *J* = 7.2 Hz, 1H), 5.61 (s, 2H), 3.14–3.04 (m, 2H), 1.29 (d, *J* = 6.8 Hz, 6H), 1.01 (d, *J* = 6.8 Hz, 6H). ^13^C NMR (100MHz, CDCl_3_, TMS): δ 164.4, 162.9, 162.0, 161.8, 160.4, 159.6, 147.6, 146.7, 145.5, 139.9, 137.9, 136.4, 135.4, 134.3, 131.0, 130.8, 130.7, 130.0, 129.3, 128.9, 128.2, 127.7, 127.5, 126.6, 124.9, 123.8, 123.4, 121.1, 115.4, 115.2, 115.0, 50.6, 28.7, 24.2, 23.7. ^19^F NMR (470 MHz, CDCl_3_, TMS): δ −58.19, −116.12, −116.28. FT-IR (cm^−1^): 3053 (w), 2965 (w), 2932 (w), 2876 (w), 1671 (w, *v*_C = N_), 1649 (w, *v*_C = N_), 1598 (w), 1506 (s), 1436 (w), 1392 (w), 1361 (w), 1333 (w), 1300 (w), 1228 (w), 1165 (w), 1095 (w), 1041 (w), 1014 (w), 923 (w), 876 (w), 830 (m), 785 (w), 761 (w). Anal. calcd for C_57_H_43_F_7_N_2_O·H_2_O (904.97): C, 75.65; H, 4.79; N, 3.10. Found: C, 75.37; H, 5.06; N, 3.44%.

**L4.** Using a typical procedure as described for the synthesis of **L1**, **L4** was prepared as a yellow powder (0.66 g, 38.0%). ^1^H NMR (400 MHz, CDCl_3_, TMS): δ 7.81 (d, *J* = 8.0 Hz, 1H), 7.74 (d, *J* = 8.4 Hz, 1H), 7.34 (t, *J* = 7.8 Hz, 1H), 7.07–6.93 (m, 11H), 6.86–6.82 (m, 6H), 6.66 (d, *J* = 7.2 Hz, 1H), 6.32 (t, *J* = 8.6 Hz, 4H), 5.98 (d, *J* = 6.8 Hz, 1H), 5.58 (s, 2H), 2.39 (s, 3H), 2.15 (s, 6H). ^13^C NMR (100MHz, CDCl_3_, TMS): δ 164.2, 162.9, 162.1, 161.2, 160.5, 159.6, 147.5, 146.4, 145.4, 139.9, 137.6, 136.6, 134.2, 133.4, 131.1, 131.0, 130.8, 130.7, 130.1, 129.2, 128.5, 128.1, 126.6, 124.3, 123.5, 122.4, 121.8, 120.9, 119.3, 115.4, 115.2, 115.1, 114.9, 50.9, 50.7, 20.9, 18.0. ^19^F NMR (470 MHz, CDCl_3_, TMS): δ −58.17, −116.16, −116.39. FT-IR (cm^−1^): 3043 (w), 2975 (w), 2912 (w), 1668 (w, *v*_C = N_), 1642 (w, *v*_C = N_), 1599 (w), 1506 (m), 1482 (w), 1440 (w), 1256 (w), 1224 (w), 1155 (w), 1096 (w), 1035 (w), 1013 (w), 925 (w), 831 (m), 778 (m), 732 (w). Anal. calcd for C_54_H_37_F_7_N_2_O·H_2_O (862.89): C, 75.17; H, 4.32; N, 3.25. Found: C, 75.56; H, 4.73; N, 3.09%.

**L5**. Using a typical procedure as described for the synthesis of **L1**, **L5** was prepared as a yellow powder (0.40 g, 22.5%). ^1^H NMR (400 MHz, CDCl_3_, TMS): δ 7.79 (d, *J* = 8.4 Hz, 1H), 7.70 (d, *J* = 8.4 Hz, 1H), 7.32 (t, *J* = 7.6 Hz, 1H), 7.04 (s, 2H), 7.02–6.94 (m, 9H), 6.86–6.82 (m, 6H), 6.64 (d, *J* = 7.2 Hz, 1H), 6.30 (t, *J* = 8.4 Hz, 4H), 5.87 (d, *J* = 7.2 Hz, 1H), 5.60 (s, 2H), 2.65–2.56 (m, 2H), 2.51–2.42 (m, 5H), 1.14 (t, *J* = 7.4 Hz, 6H). ^13^C NMR (100MHz, CDCl_3_, TMS): δ 164.2, 162.9, 162.1, 161.2, 160.4, 159.6, 147.5, 146.3, 145.4, 139.9, 137.6, 136.6, 134.2, 133.4, 131.1, 131.0, 130.7, 130.6, 130.1, 129.2, 129.1, 128.4, 128.0, 126.6, 124.3, 123.5, 122.4, 121.0, 115.4, 115.2, 115.0, 114.8, 50.8, 50.6, 20.9,18.0. ^19^F NMR (470 MHz, CDCl_3_, TMS): δ −58.18, −116.17, −116.38. FT-IR (cm^−1^): 3048 (w), 2965 (w), 2918 (w), 2854 (w), 1661 (w, *v*_C = N_), 1640 (w, *v*_C = N_), 1598 (w), 1505 (m), 1463 (w), 1438 (w), 1256 (w), 1217 (w), 1152 (w), 1093 (w), 1042 (w), 1013 (w), 927 (w), 864 (w), 829 (w), 786 (w). Anal. calcd for C_56_H_41_F_7_N_2_O·H_2_O (890.95): C, 75.49; H, 4.64; N, 3.14. Found: C, 75.12; H, 4.26; N, 3.54%.

### 3.3. Synthesis of Nickel Complexes (***Ni1***–***Ni5***)

**Ni1**. Under a nitrogen atmosphere, **L1** (0.10 g, 0.12 mmol), NiBr_2_(DME) (0.04 g, 0.12 mmol), dichloromethane (6 mL), and ethanol (4 mL) were stirred at ambient temperature for 12 h. The solution was concentrated, and diethyl ether (25 mL) was added to precipitate the product. It was collected by filtration and washed with excess diethyl ether (3 × 15 mL) affording **Ni1** as a red powder (0.12 g, 92.1% yield). ^1^H NMR (400 MHz, CD_2_Cl_2_, TMS): δ 28.48 (s, 6H, −CH_3_), 25.39 (s, 2H, Ar-Hm), 25.06 (s, 1H, An-H), 21.81 (s, 2H, Ar-Hm), 18.74 (s, 1H, An-H), 16.90 (s, 1H, An-H), 16.06 (s, 1H, An-H), 11.91 (broad, 0.81H, −C*H*Ph_2_F_2_), 8.16 (s, 4H, Ar-H), 7.07 (s, 4H, Ar-H), 6.31 (s, 1H, An-H), 5.46 (s, 4H, Ar-H), 5.05 (s, 1H, An-H), 1.25 (s, 4H, Ar-H), -16.17 (s, 1H, Ar-Hp). ^19^F NMR (470 MHz, CDCl_3_, TMS): δ −57.12, −115.73, −116.07. FT-IR (cm^−1^): 3071 (w), 3036 (w), 2972 (w), 2906 (w), 1649 (w, *v*_C = N_), 1623 (w, *v*_C = N_), 1601 (w), 1585 (w), 1506 (s), 1444 (w), 1421 (w), 1262 (w), 1221 (w), 1158 (m), 1096 (w), 1051 (w), 1014 (w), 959 (w), 878 (w), 834 (m), 773 (m). Anal. calcd for C_53_H_35_Br_2_F_7_N_2_NiO (1067.37): C, 59.64; H, 3.31; N, 2.62. Found: C, 60.01; H, 3.17; N, 2.35%.

**Ni2**. Using a typical procedure as described for the synthesis of **Ni1**, **Ni2** was also afforded as a red powder (0.11 g, 86.2% yield). ^1^H NMR (400 MHz, CDCl_3_, TMS): δ 28.89 (s, 2H, −C*H*_2_CH_3_), 27.44 (s, 2H, −C*H*_2_CH_3_), 25.69 (s, 1H, An-H), 25.51 (s, 2H, Ar-Hm), 22.31 (s, 2H, Ar-Hm), 19.48 (s, 1H, An-H), 17.36 (s, 1H, An-H), 16.52 (s, 1H, An-H), 13.21 (broad, 1.2H, −C*H*Ph_2_F_2_), 8.20 (s, 4H, Ar-H), 7.07 (s, 4H, Ar-H), 6.09 (s, 1H, An-H), 5.54 (s, 4H, Ar-H), 4.97 (s, 1H, An-H), 1.79 (s, 2H, Ar-H), 1.22 (s, 1H, Ar-H), 0.81 (s, 7H, Ar-H, −CH_2_C*H*_3_), −16.87 (s, 1H, Ar-Hp). ^19^F NMR (470 MHz, CDCl_3_, TMS): δ −57.09, −115.67, −116.16. FT-IR (cm^−1^): 3069 (w), 3046 (w), 2978 (w), 2942 (w), 1649 (w, *v*_C = N_), 1621 (w, *v*_C = N_), 1603 (w), 1583 (w), 1505 (s), 1440 (w), 1421 (w), 1293 (w), 1256 (w), 1217 (w), 1157 (w), 1104 (w), 1050 (w), 1014 (w), 958 (w), 877 (w), 831 (m), 771 (w), 729 (w). Anal. calcd for C_55_H_39_Br_2_F_7_N_2_NiO (1095.42): C, 60.31; H, 3.59; N, 2.56. Found: C, 60.11; H, 3.46; N, 2.78%.

**Ni3**. Using a typical procedure as described for the synthesis of **Ni1**, **Ni3** was also afforded as a red powder (0.11 g, 84.9% yield). ^1^H NMR (400 MHz, CDCl_3_, TMS): δ 26.25 (s, 1H, An-H), 25.16 (s, 2H, Ar-Hm), 22.75 (s, 2H, Ar-Hm), 2.10 (s, 1H, An-H), 17.89 (s, 1H, An-H), 16.72 (s, 1H, An-H), 13.80 (broad, 2H, −C*H*Ph_2_F_2_), 8.14 (s, 4H, Ar-H), 7.02 (s, 4H, Ar-H), 6.85 (s, 1H, An-H), 5.68 (s, 6H, Ar-H), 4.97 (s, 1H, An-H), 1.87 (s, 7H, Ar-H, −CH(C*H*_3_)_2_), 1.69 (s, 6H, −CH(C*H*_3_)_2_), 1.30 (s, 1H, Ar-H), 1.03 (s, 2H, −C*H*(CH_3_)_2_), −16.41 (s, 1H, Ar-Hp). ^19^F NMR (470 MHz, CDCl_3_, TMS): δ −57.19, −115.91, −116.09. FT-IR (cm^−1^): 3072 (w), 3038 (w), 2969 (w), 2913 (w), 1645 (w, *v*_C = N_), 1620 (w, *v*_C = N_), 1601 (w), 1582 (w), 1506 (s), 1442 (w), 1421 (w), 1390 (w), 1364 (w), 1328 (w), 1298 (w), 1261 (w), 1220 (w), 1159 (w), 1097 (w), 1050 (w), 1014 (w), 958 (w), 876 (w), 832 (m), 772 (w), 734 (w). Anal. calcd for C_57_H_43_Br_2_F_7_N_2_NiO (1123.47): C, 60.94; H, 3.86; N, 2.49. Found: C, 60.73; H, 3.52; N, 2.57%.

**Ni4**. Using a typical procedure as described for the synthesis of **Ni1**, **Ni4** was also afforded as a red powder (0.11 g, 87.8% yield). ^1^H NMR (400 MHz, CDCl_3_, TMS): δ 34.47 (s, 3H, Ar–p–CH_3_), 29.31 (s, 6H, -CH_3_), 25.92 (s, 1H, An-H), 25.31 (s, 2H, Ar-Hm), 22.07 (s, 2H, Ar-Hm), 19.14 (s, 1H, An-H), 17.23 (s, 1H, An-H), 16.40 (s, 1H, An-H), 12.71 (broad, 0.99H, −C*H*Ph_2_F_2_), 8.18 (s, 4H, Ar-H), 7.07 (s, 4H, Ar-H), 6.20 (s, 1H, An-H), 5.47 (s, 4H, Ar-H), 4.92 (s, 1H, An-H), 1.26 (s, 2H, Ar-H), 1.07 (s, 2H, Ar-H). ^19^F NMR (470 MHz, CDCl_3_, TMS): δ −57.15, −115.78, −116.10. FT-IR (cm^−1^): 3069 (w), 2991 (w), 2911 (w), 1643 (w, *v*_C = N_), 1624 (w, *v*_C = N_), 1602 (w), 1583 (w), 1505 (s), 1443 (w), 1269 (m), 1217 (m), 1191 (w), 1155 (m), 1096 (w), 1048 (w), 1015 (w), 962 (w), 877 (w), 832 (m), 777 (w). Anal. calcd for C_54_H_37_Br_2_F_7_N_2_NiO (1081.39): C, 59.98; H, 3.45; N, 2.59. Found: C, 60.26; H, 3.58; N, 2.27%.

**Ni5**. Using a typical procedure as described for the synthesis of **Ni1**, **Ni5** was also afforded as a red powder (0.12 g, 90.5% yield). ^1^H NMR (400 MHz, CDCl_3_, TMS): δ 34.75 (s, 3H, Ar–p–CH_3_), 28.61 (s, 2H, −C*H*_2_CH_3_), 27.53 (s, 2H, −C*H*_2_CH_3_), 26.25 (s, 1H, An-H), 25.19 (s, 2H, Ar-Hm), 22.29 (s, 2H, Ar-Hm), 19.29 (s, 1H, An-H), 17.30 (s, 1H, An-H), 16.54 (s, 1H, An-H), 13.02 (broad, 1.2H, −C*H*Ph_2_F_2_), 8.24 (s, 4H, Ar-H), 7.08 (s, 4H, Ar-H), 6.16 (s, 1H, An-H), 5.53 (s, 4H, Ar-H), 4.90 (s, 1H, An-H), 1.22 (s, 3H, Ar-H), 0.87 (s, 7H, Ar-H, −CH_2_C*H*_3_). ^19^F NMR (470 MHz, CDCl_3_, TMS): δ −58.16, −115.86, −116.09. FT-IR (cm^−1^): 3079 (w), 2975 (w), 2935 (w), 2873 (w), 1644 (w, *v*_C = N_), 1618 (w, *v*_C = N_), 1602 (w), 1583 (w), 1504 (s), 1444 (w), 1383 (w), 1296 (w), 1252 (w), 1220 (w), 1174 (w), 1157 (w), 1098 (w), 1054 (w), 1015 (w), 960 (w), 868 (w), 831 (m), 773 (m), 731 (w). Anal. calcd for C_56_H_41_Br_2_F_7_N_2_NiO (1109.45): C, 60.63; H, 3.73; N, 2.53. Found: C, 60.48; H, 3.61; N, 2.88%.

## 4. Conclusions

In this study, a novel series of asymmetric 1,2-bis(imine)acenaphthene nickel(II) complexes (**Ni1**–**Ni5**) featuring para-trifluoromethoxy and distal-fluoro-benzhydryl groups, was developed. These dual-functionalized catalysts achieved exceptional ethylene polymerization activity (up to 22.0 × 10^6^ g PE mol^−1^(Ni) h^−1^) and outstanding thermal stability, maintaining 4.82 × 10^6^ g PE mol^−1^(Ni) h^−1^ at 80 °C. The synergistic combination of electron-withdrawing trifluoromethoxy and sterically shielding fluorobenzhydryl groups enhanced both the electrophilicity of the nickel center and resistance to thermal deactivaton, enabling efficient ethylene polymerization under industrially relevant conditions. By systematically modulating ligand architecture and co-catalyst, solvent (toluene vs. n-hexane), and polymerization parameters (temperature, time, and ethylene pressure), we achieved precise control over PE branching density and molecular weight. The strong electron-withdrawing *para*-trifluoromethoxy group facilitates ethylene insertion, thereby increasing the molecular weight of the resultant polymer. Furthermore, the bulky *ortho*-substituent promotes chain growth and chain walking while effectively suppressing chain transfer reactions, leading to the formation of polyethylenes with high molecular weight and high branching density. The resulting PEs displayed highly branching levels alongside robust mechanical properties, including tensile strengths up to 20.7 MPa and 58% elastic recovery. Comprehensive characterization of ligands, complexes, and polymers revealed structure–property relationships critical for advancing TPEs production technologies. This work underscores the potential of rationally designed nickel catalysts for high-temperature ethylene polymerization processes, offering a versatile platform to tailor polyolefin architectures for advanced elastomeric applications, where the integration of fluorine-based substituents elevates catalytic efficiency and enables scalable production of high-performance thermoplastic elastomers (TPEs) with tunable mechanical properties.

## Figures and Tables

**Table 1:** Key bond parameters for **Ni2** and **Ni4**.

	Ni2	Ni4
Bond lengths (Å)		
Ni(1)-Br(1)	2.3342 (6)	2.3253 (16)
Ni(1)-Br(2)	2.3300 (7)	2.3388 (17)
Ni(1)-N(1)	2.037 (3)	2.041 (7)
Ni(1)-N(2)	2.022 (3)	2.011 (7)
N(1)-C(12)	1.290 (4)	1.261 (11)
N(1)-C(13)	1.446 (4)	1.448 (10)
N(2)-C(1)	1.283 (5)	1.289 (11)
N(2)-C(46)	1.440 (4)	1.450 (11)
O(1)-C(45)_CF3_	1.342 (6)	1.23 (5)
Bond angles (°)		
N(1)-Ni(1)-N(2)	82.73 (12)	82.6 (3)
Br(1)-Ni(1)-Br(2)	126.05 (3)	126.56 (7)
N(1)-Ni(1)-Br(1)	110.13 (7)	109.70 (19)
N(1)-Ni(1)-Br(2)	110.20 (7)	111.2 (2)
N(2)-Ni(1)-Br(1)	110.18 (8)	107.7 (2)
N(2)-Ni(1)-Br(2)	109.08 (8)	110.3 (2)
C(45)-O(1)-C(16)	116.3 (4)	125 (3)

**Table 2:** Ethylene polymerization results with **Ni1** using various alkylalumimum co-catalysts *^a^*.

Run	Co-Catalyst	Al/Ni	PE (g)	Activity *^b^*	M_w_ *^c^*	M_w_/M_n_ *^c^*	T_m_ *^d^* (°C)
1	MAO	2000	3.36	6.72	4.51	3.09	81.7
2	MMAO	2000	4.34	8.68	6.85	3.52	97.0
3	EtAlCl_2_	300	6.59	13.2	5.96	3.49	66.4
4	EASC	300	4.39	8.78	7.54	2.48	99.6
5	Et_2_AlCl	300	3.64	7.28	5.80	4.03	80.9

*^a^* Conditions: 1.0 μmol **Ni1**, 100 mL of toluene, 10 atm C_2_H_4_, 30 °C, 30 min; *^b^* 10^6^ g (PE) mol^−1^ (Ni) h^−1^; *^c^* M_w_: ×10^5^ g mol^−1^, M_w_ and M_w_/M_n_ determined by GPC; *^d^* Determined by DSC.

**Table 3:** Ethylene polymerization with **Ni1**–**Ni5**/EtAlCl_2_ *^a^*.

Run	Precat.	Al/Ni	T (°C)	T (min)	PE (g)	Activity *^b^*	M_w_ *^c^*	M_w_/M_n_ *^c^*	T_m_ *^d^* (°C)
1	**Ni1**	300	30	30	6.59	13.2	5.96	3.49	66.4
2	**Ni1**	300	40	30	7.11	14.2	4.78	3.55	61.6
3	**Ni1**	300	50	30	4.77	9.54	4.26	2.70	45.5
4	**Ni1**	300	60	30	3.84	7.68	3.21	1.73	36.8
5	**Ni1**	300	70	30	2.42	4.84	2.64	2.51	35.8
6	**Ni1**	300	80	30	1.38	2.76	2.26	2.78	16.8
7	**Ni1**	100	40	30	5.37	10.7	4.18	2.61	58.6
8	**Ni1**	150	40	30	6.30	12.6	4.43	2.74	66.5
9	**Ni1**	200	40	30	8.59	17.2	4.59	2.29	59.4
10	**Ni1**	250	40	30	8.07	16.1	4.88	3.23	54.2
11	**Ni1**	200	40	5	1.63	19.6	2.78	3.00	62.3
12	**Ni1**	200	40	15	4.57	18.3	3.68	3.08	67.3
13	**Ni1**	200	40	45	10.6	14.1	4.86	2.16	56.3
14	**Ni1**	200	40	60	11.8	11.8	5.08	2.88	62.8
15 *^e^*	**Ni1**	200	40	30	3.58	7.16	3.73	2.59	55.4
16 *^f^*	**Ni1**	200	40	30	0.74	1.48	2.33	1.75	- *^g^*
17	**Ni2**	200	40	30	5.16	10.3	4.21	2.70	59.0
18	**Ni3**	200	40	30	3.37	6.74	6.68	2.90	51.1
19	**Ni4**	200	40	30	7.63	15.3	4.23	3.07	63.8
20	**Ni5**	200	40	30	6.98	14.0	3.57	2.22	58.7

*^a^* Conditions: 1.0 μmol pre-catalyst, 100 mL of toluene, 10 atm C_2_H_4_; *^b^* 10^6^ g (PE) mol^−1^ (Ni) h^−1^; *^c^* M_w_: ×10^5^ g mol^−1^, M_w_ and M_w_/M_n_ determined by GPC; *^d^* Determined by DSC; *^e^* 5 atm C_2_H_4_; *^f^* 1 atm C_2_H_4_; *^g^* Broad and weak endotherms, amorphous polymers.

**Table 4:** Ethylene polymerization with **Ni1**–**Ni5**/EASC *^a^*.

Run	Precat.	Al/Ni	T (°C)	T (min)	PE (g)	Activity *^b^*	M_w_ *^c^*	M_w_/M_n_ *^c^*	T_m_ *^d^* (°C)
1	**Ni1**	300	30	30	4.39	8.78	7.54	2.48	99.6
2	**Ni1**	300	40	30	10.7	21.4	6.41	3.59	58.7
3	**Ni1**	300	50	30	8.41	16.8	5.92	4.62	51.9
4	**Ni1**	300	60	30	6.18	12.4	4.71	2.64	51.6
5	**Ni1**	300	70	30	3.04	6.08	3.61	3.97	39.8
6	**Ni1**	300	80	30	2.41	4.82	3.44	3.08	32.9
7	**Ni1**	250	40	30	9.74	19.5	5.90	3.69	65.2
8	**Ni1**	350	40	30	11.0	22.0	6.15	3.50	59.2
9	**Ni1**	400	40	30	9.85	19.7	7.12	3.40	65.2
10	**Ni1**	450	40	30	8.83	17.7	7.16	3.80	56.7
11	**Ni1**	350	40	5	2.77	33.2	3.59	3.12	79.9
12	**Ni1**	350	40	15	6.47	25.9	5.10	3.04	83.9
13	**Ni1**	350	40	45	12.8	17.1	6.55	5.08	94.4
14	**Ni1**	350	40	60	13.4	13.4	7.78	3.09	72.7
15 *^e^*	**Ni1**	350	40	30	5.18	10.4	4.29	2.62	72.5
16 *^f^*	**Ni1**	350	40	30	0.86	1.72	3.70	2.17	- *^g^*
17	**Ni2**	350	40	30	7.41	14.8	5.10	3.32	81.2
18	**Ni3**	350	40	30	6.28	12.6	5.06	3.32	59.8
19	**Ni4**	350	40	30	7.11	14.2	3.84	2.87	78.2
20	**Ni5**	350	40	30	8.61	17.2	3.47	3.74	68.3

*^a^* Conditions: 1.0 μmol pre-catalyst, 100 mL of toluene, 10 atm C_2_H_4_; *^b^* 10^6^ g (PE) mol^−1^ (Ni) h^−1^; *^c^* M_w_: ×10^5^ g mol^−1^, M_w_ and M_w_/M_n_ determined by GPC; *^d^* Determined by DSC; *^e^* 5 atm C_2_H_4_; *^f^* 1 atm C_2_H_4_; *^g^* Broad and weak endotherms, amorphous polymers.

**Table 5:** Solvent effect on **Ni1**/EASC performance *^a^*.

Run	Al/Ni	T (°C)	T (min)	PE (g)	Activity *^b^*	M_w_ *^c^*	M_w_/M_n_ *^c^*	T_m_ *^d^* (°C)
1	350	40	30	1.23	2.46	8.24	3.65	116.8
2	450	40	30	5.22	10.4	7.36	3.60	114.7
3	500	40	30	6.16	12.3	6.63	3.52	104.9
4	550	40	30	6.87	13.7	6.09	3.60	110.3
5	600	40	30	3.79	7.58	7.63	3.80	119.7
6	550	30	30	4.00	8.00	11.1	2.33	118.4
7	550	50	30	6.01	12.0	3.05	3.31	107.4

*^a^* Conditions: 1.0 μmol **Ni1**, 100 mL of hexane, 10 atm C_2_H_4_; *^b^* 10^6^ g (PE) mol^−1^ (Ni) h^−1^; *^c^* M_w_: ×10^5^ g mol^−1^, M_w_ and M_w_/M_n_ determined by GPC; *^d^* Determined by DSC.

**Table 7:** Stress strain properties of the selected PE samples.

PE Sample	*T*_m_ (°C) *^a^*	*M*_w_^ *b*^	Branches/1000 C’s	*Χ*_c_ *^a^* (%)	Stress (MPa) *^c^*	Strain (%) *^c^*
PE-40_EADC/300/T_	61.6	4.78	96.5	23.2	16.4	585
PE-40_EASC/300/T_	58.7	6.41	90.5	22.3	15.3	482
PE-60_EASC/300/T_	51.6	4.71	107	17.0	13.7	572
PE-40_EASC/450/T_	56.7	7.16	102	19.5	16.8	237
PE-40_EASC/450/H_	114.7	7.36	52.6	43.4	20.7	439

*^a^* Determined by DSC, *Χ*_c_ = 100 × ΔH_f_ (*T*_m_)/[ΔH^°^_f_ (*T*^°^_m_)], where ΔH^°^_f_ (*T*^°^_m_) = 248.3 J g^−1^; *^b^ M*_w_ (×10^5^ g mol^−1^), determined by GPC; *^c^* Determined using a universal testing instrument.

## Data Availability

CCDC reference numbers 2451477 and 2451478 contain supplementary crystallographic information for **Ni2** and **Ni4** (see Supplementary Materials), and the data presented in this study are available in this article.

## Supplementary Materials

The following supporting information can be downloaded at: https://www.mdpi.com/article/10.3390/molecules30132706/s1. General Considerations [48], Table S1: Crystal data and structure refinement for **Ni2** and **Ni4** [59,60,61]; Figure S1–S15: ^1^H/^13^C/^19^F NMR spectra of **L1**–**L5**; Figure S16–S25: ^1^H/^19^F NMR spectra of **Ni1**–**Ni5**; Figure S26: GPC traces of the PE’s obtained using **Ni1**/EtAlCl_2_; Figure S27–S31: ^13^C NMR spectra of the selected PEs. References [59,60,61] are cited in the Supplementary Materials.
