# Supplementary material for: Trifluoromethoxy- and Fluorobenzhydryl-Tuned Nickel Catalysts for Polyethylene Elastomers
Source: Molecules. 2025 Jun 23;30(13):2706. doi: 10.3390/molecules30132706 (PMC12251286; doi:10.3390/molecules30132706)
Supplement: Supplementary file 1 [file molecules-30-02706-s001.zip › molecules-3677418-supplementary.pdf]

Supporting Information

for

Trifluoromethoxy and Fluorobenzhydryl Tuned Nickel Catalysts for Polyethylene Elastomers

Ming Liu [1], Min Sun [1], Yanping Ma[2,*], Yizhou Wang [2], Mingfeng Li [1,*] and Wen-Hua Sun [2,*]

[1] State Key Laboratory of Petroleum Molecular & Process Engineering, SINOPEC Research Institute of
Petroleum Processing Co., Ltd., Beijing 100083, China; liuming.ripp@sinopec.com (M.L.); sunmin.ripp@sinopec.com (M.S.)

[2] Key Laboratory of Engineering Plastics, Beijing National Laboratory for Molecular Sciences, Institute of Chemistry Chinese Academy of Sciences, Beijing 100190, China; wangyizhou13@iccas.ac.cn

* Correspondence: myanping@iccas.ac.cn (Y.M); limf.ripp@sinopec.com (M.L.); whsun@iccas.ac.cn (W.-H.S.)

Table of Contents Page

1	General Considerations	S2
2	General Ethylene Polymerization Procedures	S2
3	X-ray Crystallographic Studies	S3
4	$^{1}H/^{13}C/^{19}F$ NMR spectra of **L1** – **L5**	S4
5	$^{1}H/^{19}F$ NMR spectra of **Ni1** – **Ni5**	S12
6	GPC traces of the PE's obtained using **Ni1**/$EtAlCl_2$	S17
7	^{13}C NMR spectra of the polyethylenes	S18
8	References	S23

1. General Considerations

All air- or moisture-sensitive compounds were handled under a nitrogen atmosphere either in a nitrogen-filled glovebox or using standard Schlenk techniques. Solvents (toluene and n-hexane) were dried over sodium and distilled under nitrogen for 8 hours prior to use. Methylaluminoxane (1.30 M solution in toluene) and modified methylaluminoxane (1.93 M solution in n-heptane) were obtained from Anhui Botai Electronic Materials Co., while Et_2AlCl (diethylaluminum chloride), EASC (ethylaluminum sesquichloride, $Et_3Al_2Cl_3$), and $EtAlCl_2$ (ethylaluminum dichloride, 40 % solution in n-hexane) were sourced from Yantai Lianli Chemical Co. High-purity ethylene was purchased from Beijing Yanshan Petrochemical Corp. and used as received. Other reagents were purchased from Aldrich, Acros, or local suppliers. The compound 2,6-bis(bis(4-fluorophenyl)methyl)-4-(trifluoromethoxy) aniline was prepared following a literature procedure[1].

FT-IR spectra were recorded on a PerkinElmer System 2000 spectrometer, and elemental analyses were determined using a Flash EA 1112 microanalyzer. ^{1}H and ^{13}C NMR spectra of all new compounds were obtained on a Bruker DMX 400 MHz instrument at room temperature using $CDCl_3$ (CD_2Cl_2 for **Ni1**, due to solubility issues) as the solvent and TMS as the internal standard. ^{19}F NMR spectra were recorded on a Bruker AVANCE III 500 MHz instrument at ambient temperature and referenced to external CF_3COOH. The melting temperatures (T_m) of the polyethylene samples were measured using a PerkinElmer TA-Q2000 DSC analyzer under nitrogen. The following procedure was employed: a weighed sample (4.0–6.0 mg) was heated to 160 °C at a rate of 20 °C min^{-1}, held for 5 minutes at 160 °C to erase thermal history, and then cooled to −20 °C at 20 °C min^{-1}. Gel permeation chromatography (GPC) was performed on the polyethylenes using an Agilent PLGPC 220 instrument equipped with a refractive index (RI) detector operating at 150 °C, with 1,2,4-trichlorobenzene as the solvent. For the ^{1}H and ^{13}C NMR spectra of the polyethylenes, a weighed amount of polyethylene (20–40 mg) was first dissolved in 1,2-dichlorobenzene-d_4 (1 mL) at elevated temperature, with TMS as the internal standard. The spectra were recorded on a Bruker AVANCE III 500 MHz instrument. The operating conditions for the ^{1}H NMR spectra were as follows: spectral width 14.9701 kHz, acquisition time 2.1889 s, relaxation delay 1.0 s, and approximately 64 scans. The operating conditions for the ^{13}C NMR spectra were: spectral width 18.8324 kHz, acquisition time 0.87 s, relaxation delay 2.0 s, and approximately 1024 scans.

2. General Ethylene Polymerization Procedures

(a) Polymerization at 5 or 10 atm C_2H_4:

Polymerizations were performed at 5 or 10 atm of ethylene in a 250 mL stainless-steel autoclave, equipped with a mechanical stirrer and a pressure/temperature control system. At the desired temperature, a 50 mL solution of the precatalyst in toluene or hexane was injected into the autoclave, followed by an additional 25 mL of solvent to rinse

the remaining precatalyst into the autoclave. The pre-measured aluminum activator was then added, and another 25 mL of solvent was introduced to complete the addition. The autoclave was pressurized to the desired ethylene pressure, and stirring was started. After the specified reaction time, the ethylene pressure was released, and the reaction was quenched by adding 10% hydrochloric acid in ethanol. The resulting polymer was collected, washed with ethanol, dried under reduced pressure at 60°C, and weighed.

(b) **Polymerization at 1 atm C_2H_4**:

A Schlenk vessel containing a stir bar was first evacuated and then backfilled with ethylene. The precatalyst was introduced, followed by 30 mL of freshly distilled toluene and the required amount of activator ($EtAlCl_2$ or EASC). The reaction mixture was stirred under 1 atm ethylene for 30 minutes. Following the reaction, the pressure was released, and the polymerization was quenched with 10% hydrochloric acid in ethanol. The polymer was then collected, washed with ethanol, dried under reduced pressure at 60°C, and weighed.

3. X-ray Diffraction Studies

X-ray quality crystals of **Ni2** and **Ni4** were obtained by diffusing hexane onto a dichloromethane solution containing the corresponding complex. A suitable crystal from each sample was selected and mounted on an XtaLAB Synergy R diffractometer equipped with a HyPix detector, using graphite-monochromated Cu-Kα radiation (λ = 1.54184 Å) and a nitrogen cold stream. The crystal was maintained at 170 K during data collection. The structures were solved using Olex2[2] with the ShelXT[3] structure solution program employing Intrinsic Phasing, and refined with the ShelXL[4] refinement package using Least Squares minimization. The details of the X-ray structure determination and refinement are summarized in Table S1.

Table S1 Crystallographic data and structure refinement for **Ni2** and **Ni4**

	**Ni2**	**Ni4**
CCDC numbers	2451477	2451478
Empirical formula	$C_{55}H_{39}Br_2F_7N_2NiO$	$C_{54}H_{37}Br_2F_7N_2NiO$
Formula weight	1095.41	1081.38
Temperature/K	169.99(11)	169.98(11)
Crystal system	mon^{oC}linic	mon^{oC}linic
Space group	Cc	Cc
a/Å	10.56620(10)	18.1177(4)
b/Å	19.4791(2)	10.7688(4)
c/Å	25.2486(2)	29.3389(9)
α/°	90	90
β/°	91.7130(10)	101.290(3)
γ/°	90	90
Volume/Å^3	5194.35(8)	5613.4(3)
Z	4	4
ρ_{calc} (g/cm^3)	1.401	1.280
μ/mm^{-1}	2.885	2.663
F(000)	2208.0	2176.0

Crystal size/mm^3	0.2 × 0.1 × 0.02	0.2 × 0.05 × 0.01
Radiation	Cu Kα (λ = 1.54184)	Cu Kα (λ = 1.54184)
2Θ range for data collection/°	7.006 to 150.394	6.144 to 150.784
Index ranges	$-12 \le h \le 13$, $-24 \le k \le 23$, $-31 \le l \le 31$	$-21 \le h \le 22$, $-13 \le k \le 12$, $-36 \le l \le 25$
Reflections collected	20237	21088
Independent reflections	6873 [R_{int} = 0.0214, R_{sigma} = 0.0222]	6368 [R_{int} = 0.0541, R_{sigma} = 0.0429]
Data/restraints/parameters	6873/16/615	6368/231/662
Goodness-of-fit on F^2	1.055	1.056
Final R indexes [I>=2σ (I)]	R_1 = 0.0351, wR_2 = 0.0933	R_1 = 0.0710, wR_2 = 0.1907
Final R indexes (all data)	R_1 = 0.0360, wR_2 = 0.0941	R_1 = 0.0783, wR_2 = 0.1979
Largest diff. peak/hole (e Å^{-3})	1.87/-0.44	1.32/-1.10
Flack parameter	0.022(10)	0.14(3)

4. ^{1}H/^{13}C/^{19}F NMR spectra of L1 – L5

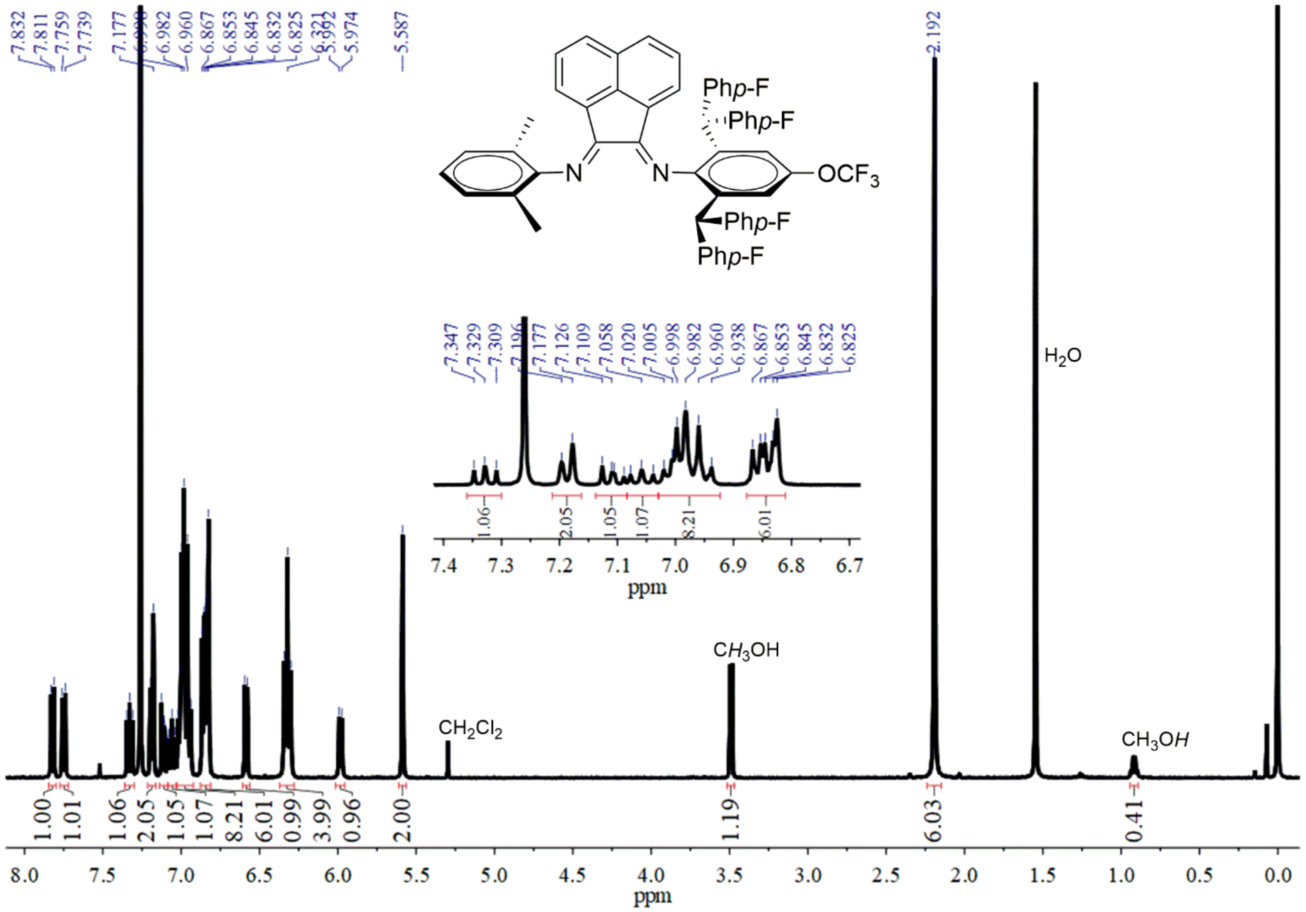

Figure S1 ^{1}H NMR spectrum of **L1** in $CDCl_3$ at room temperature.

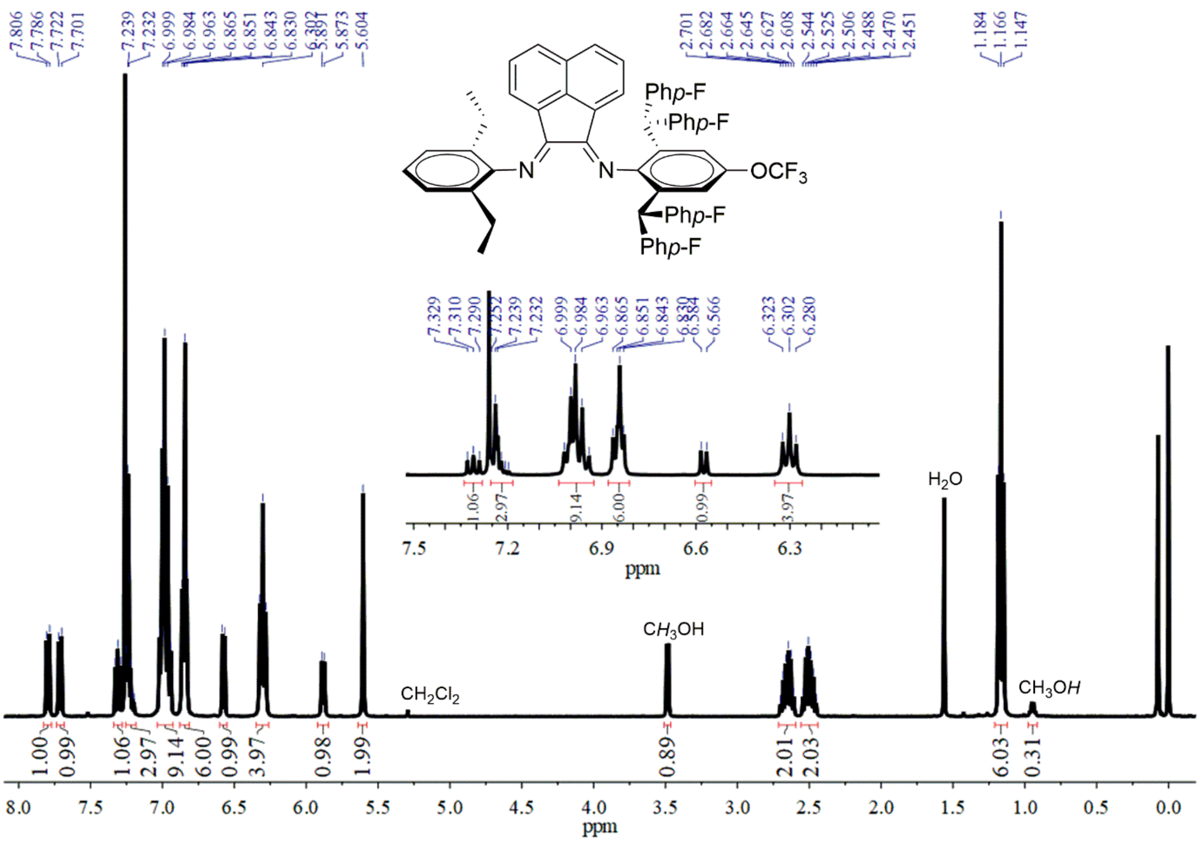

Figure S2 ^{1}H NMR spectrum of **L2** in $CDCl_3$ at room temperature.

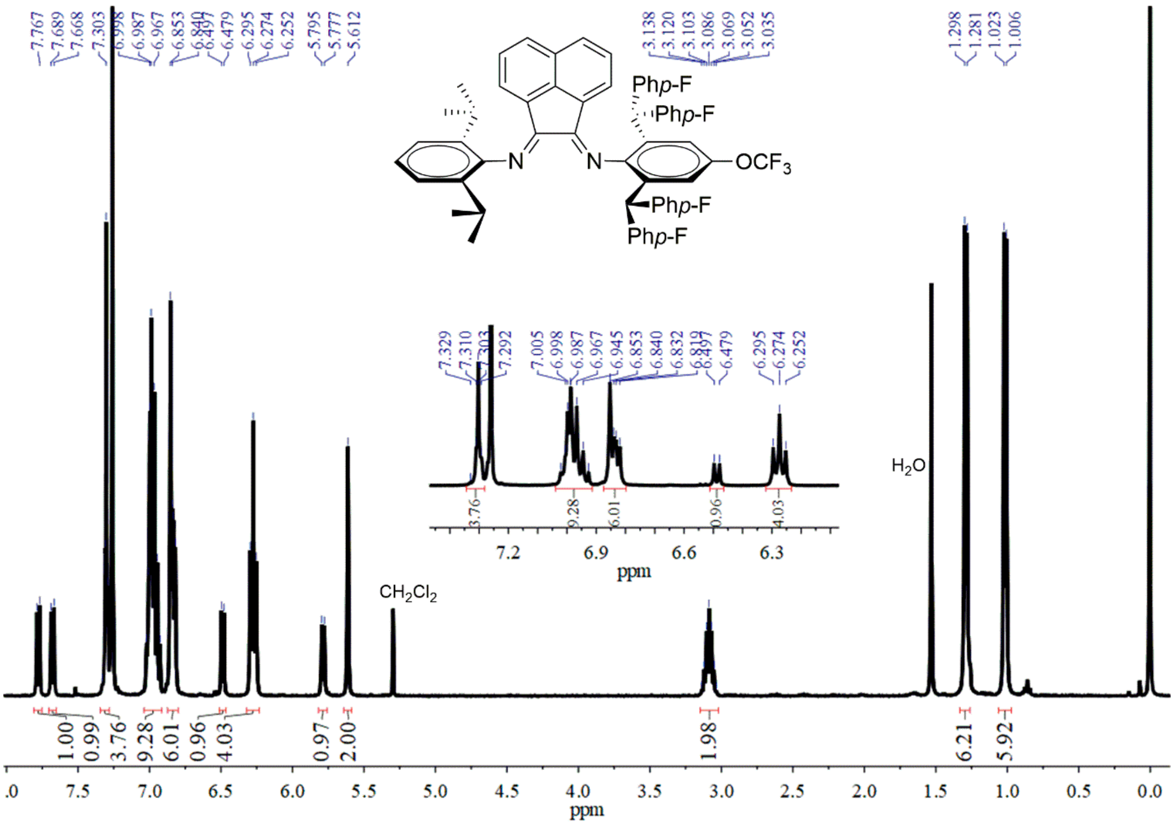

Figure S3 ^{1}H NMR spectrum of **L3** in $CDCl_3$ at room temperature.

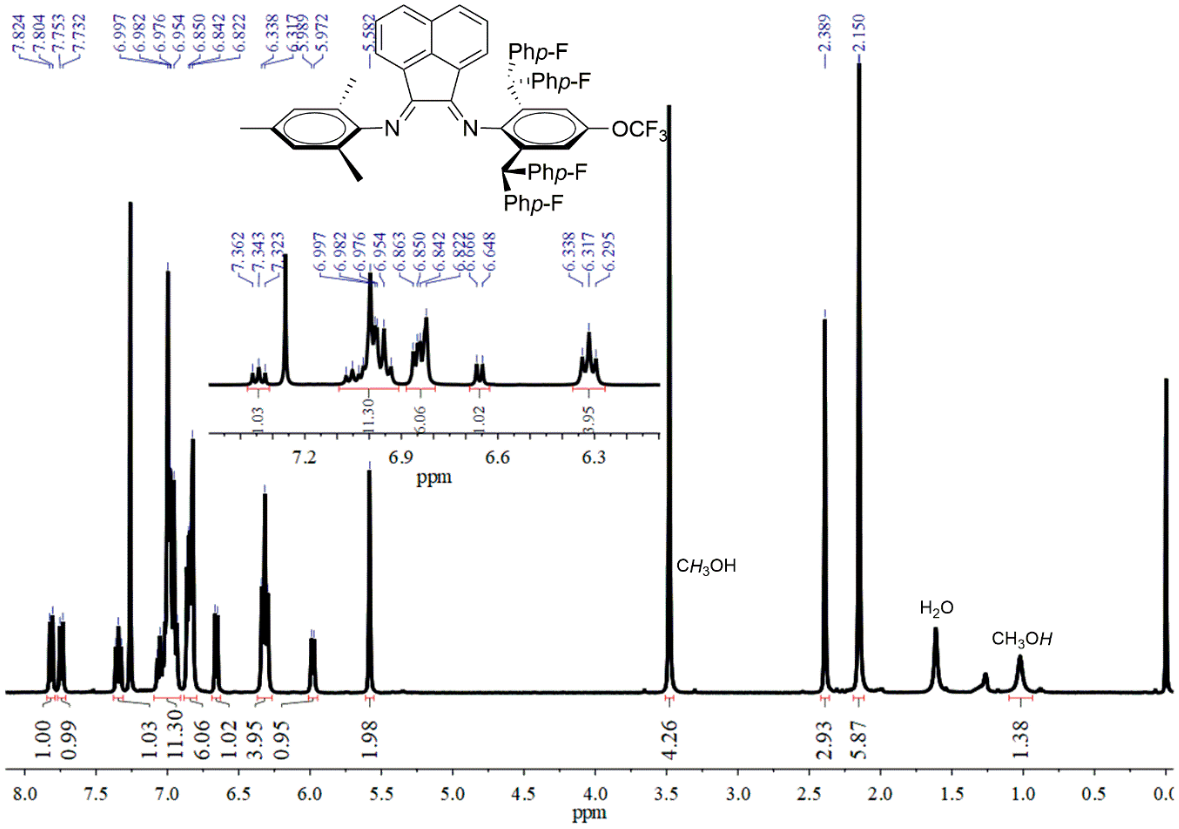

Figure S4 ^{1}H NMR spectrum of **L4** in $CDCl_3$ at room temperature.

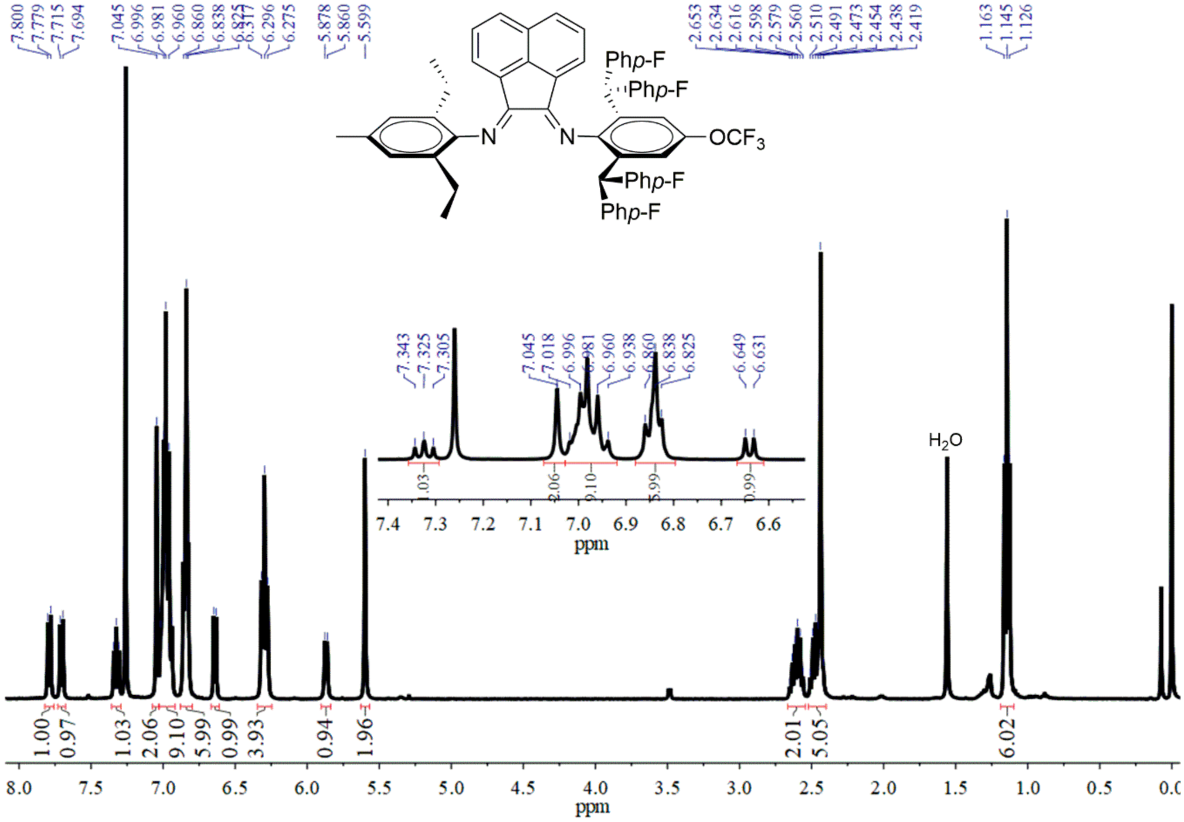

Figure S5 ^{1}H NMR spectrum of **L5** in $CDCl_3$ at room temperature.

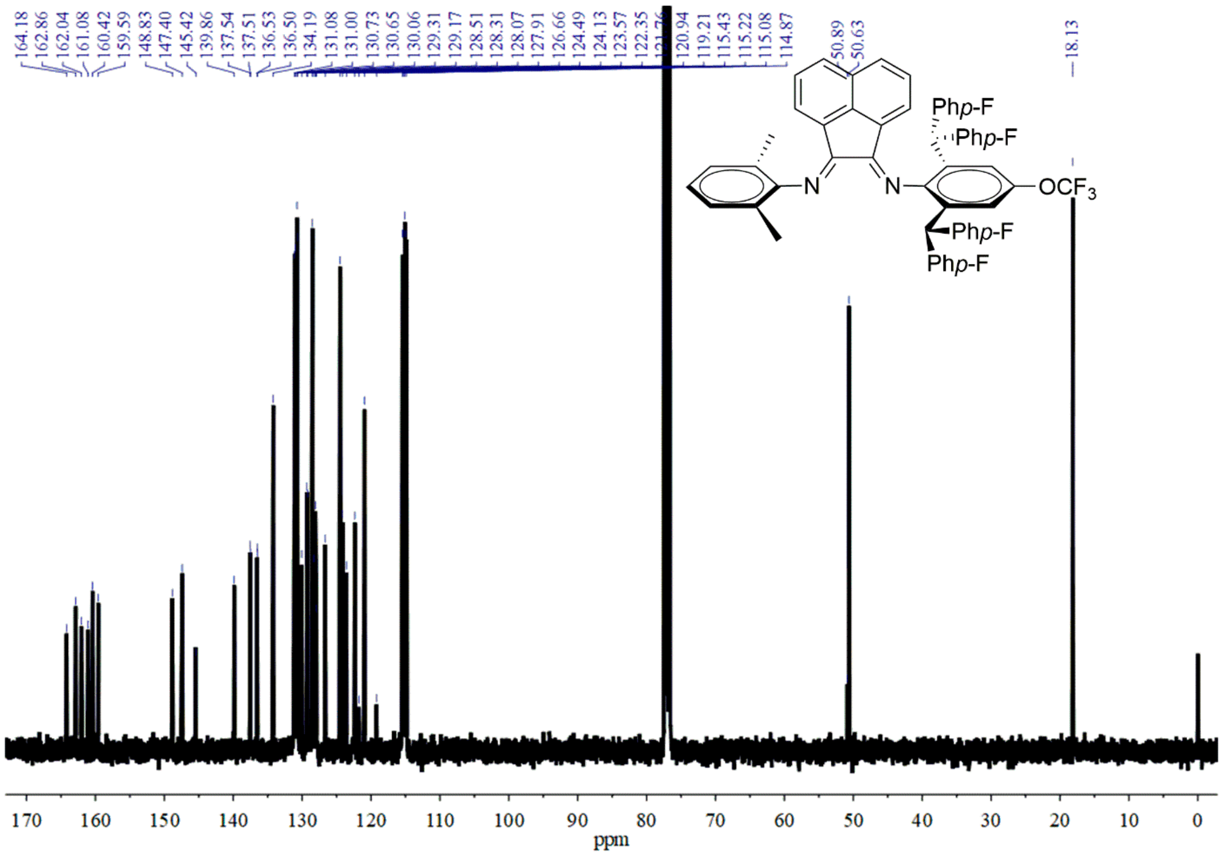

Figure S6 ^{13}C NMR spectrum of **L1** in $CDCl_3$ at room temperature.

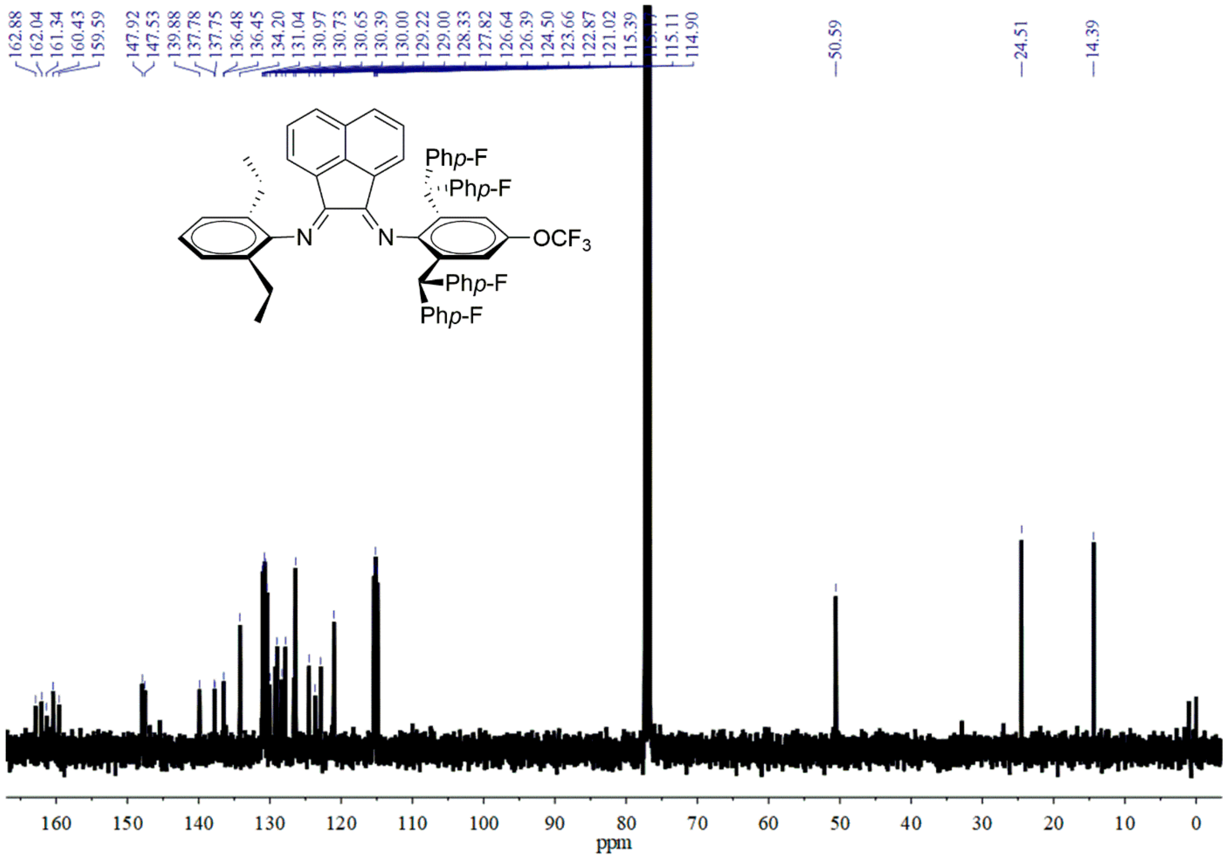

Figure S7 ^{13}C NMR spectrum of **L2** in $CDCl_3$ at room temperature.

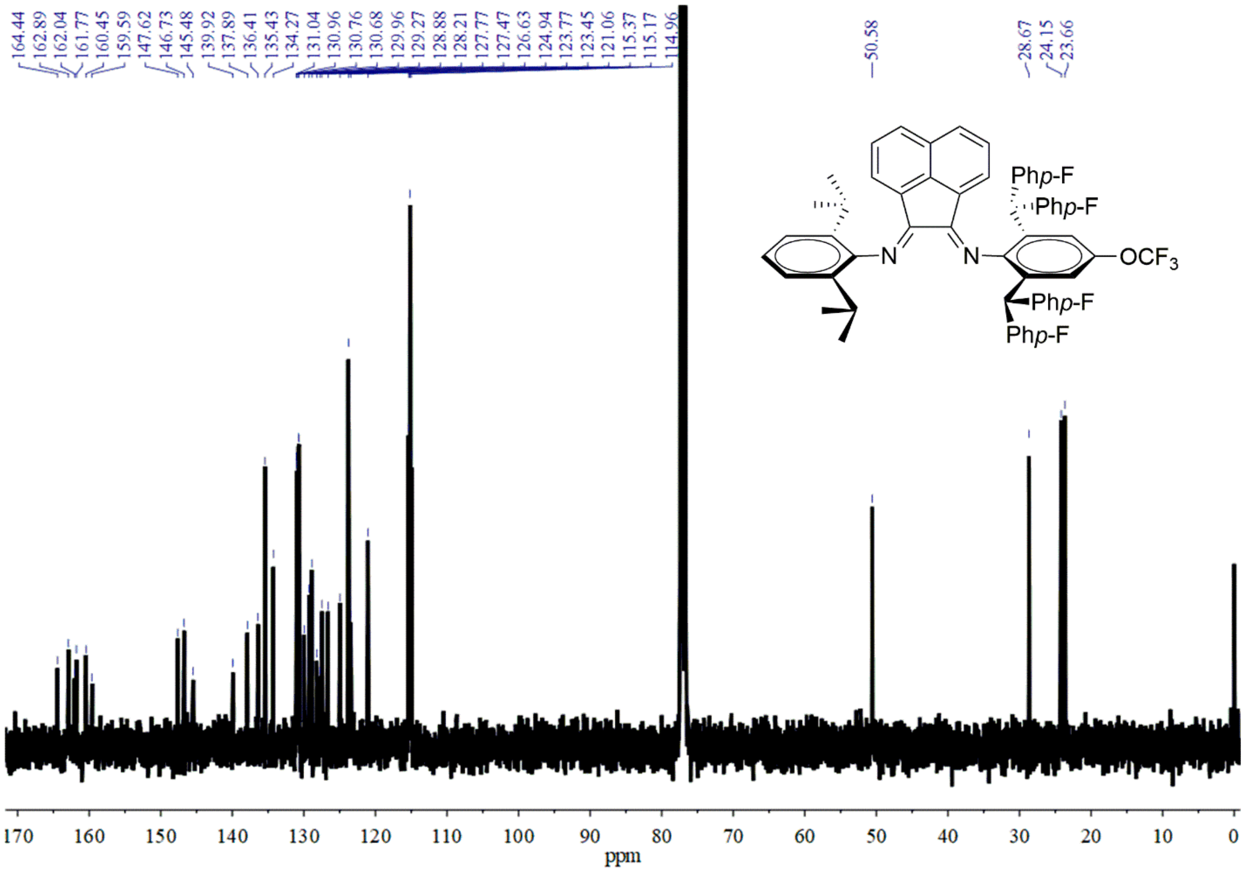

Figure S8 ^{13}C NMR spectrum of **L3** in $CDCl_3$ at room temperature.

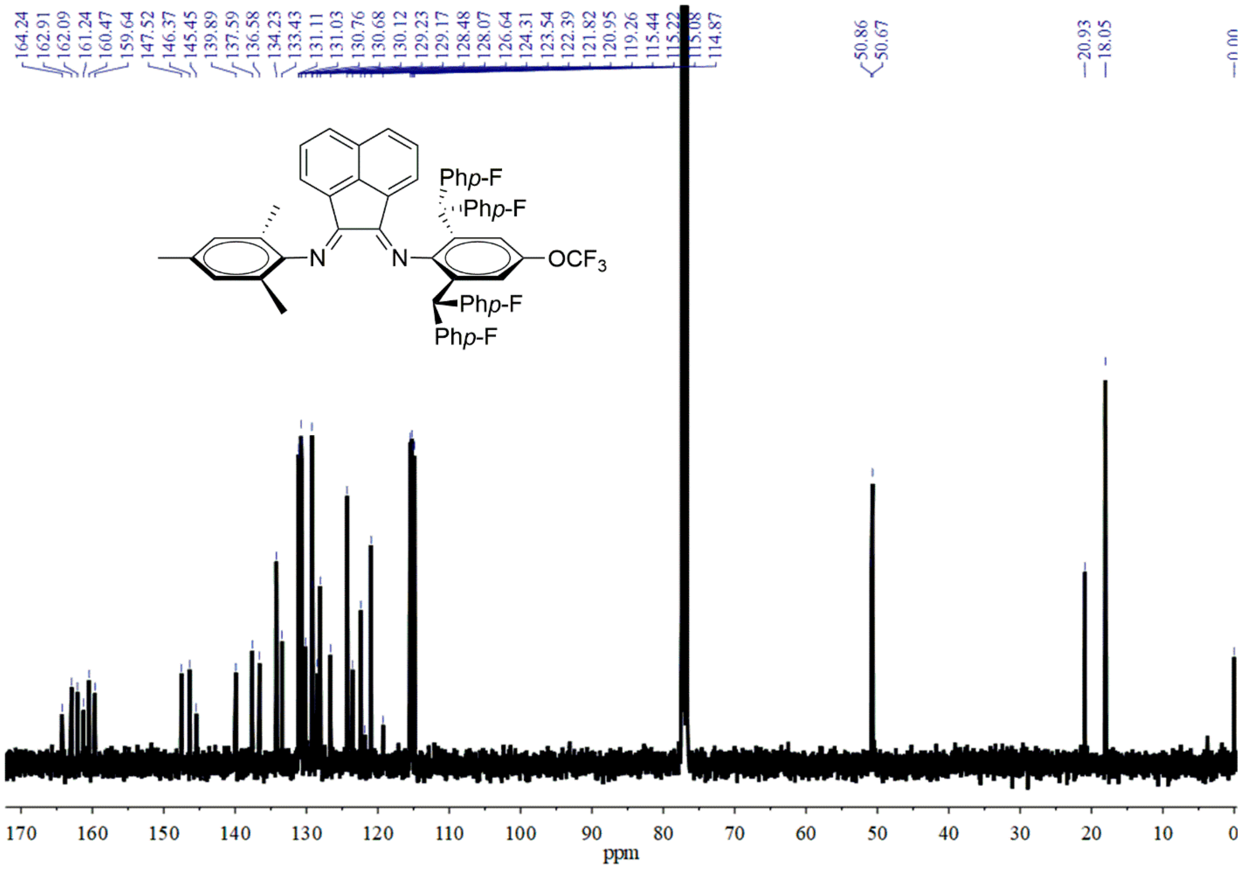

Figure S9 ^{13}C NMR spectrum of **L4** in $CDCl_3$ at room temperature.

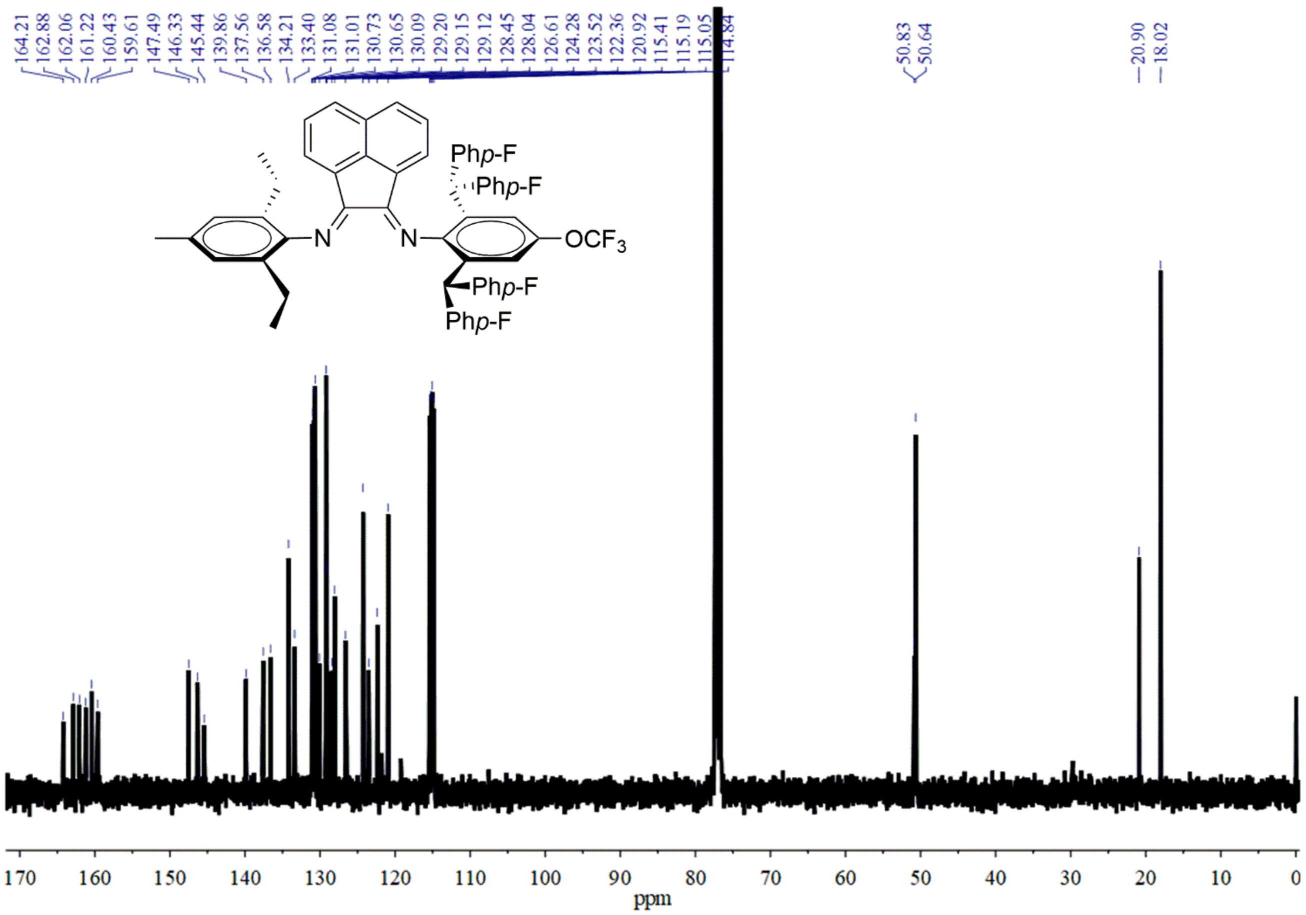

Figure S10 ^{13}C NMR spectrum of **L5** in $CDCl_3$ at room temperature.

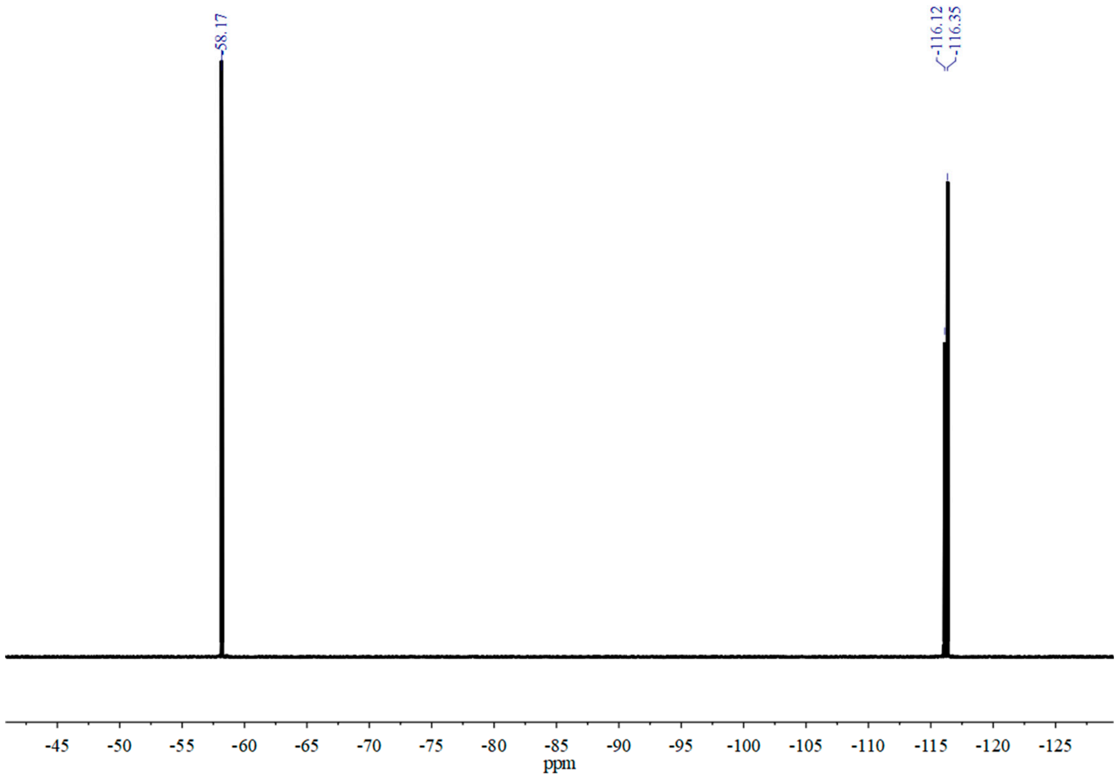

Figure S11 ^{19}F NMR spectrum of **L1** in $CDCl_3$ at room temperature.

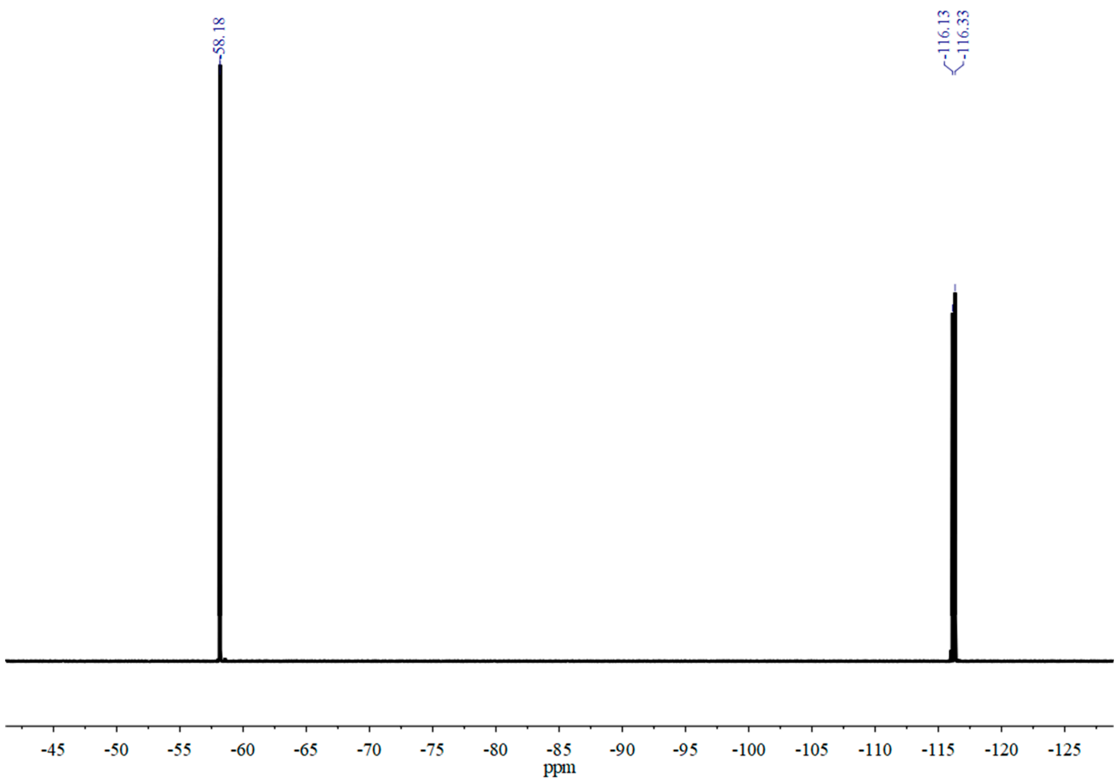

Figure S12 ^{19}F NMR spectrum of **L2** in $CDCl_3$ at room temperature.

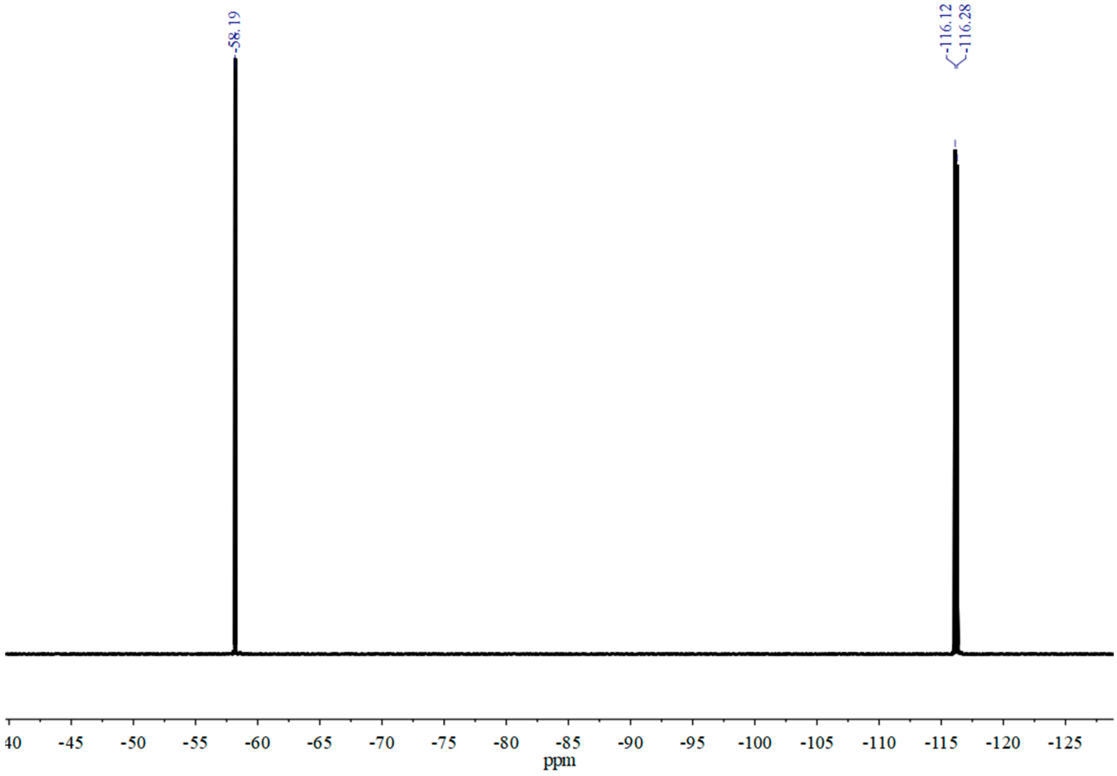

Figure S13 ^{19}F NMR spectrum of **L3** in $CDCl_3$ at room temperature.

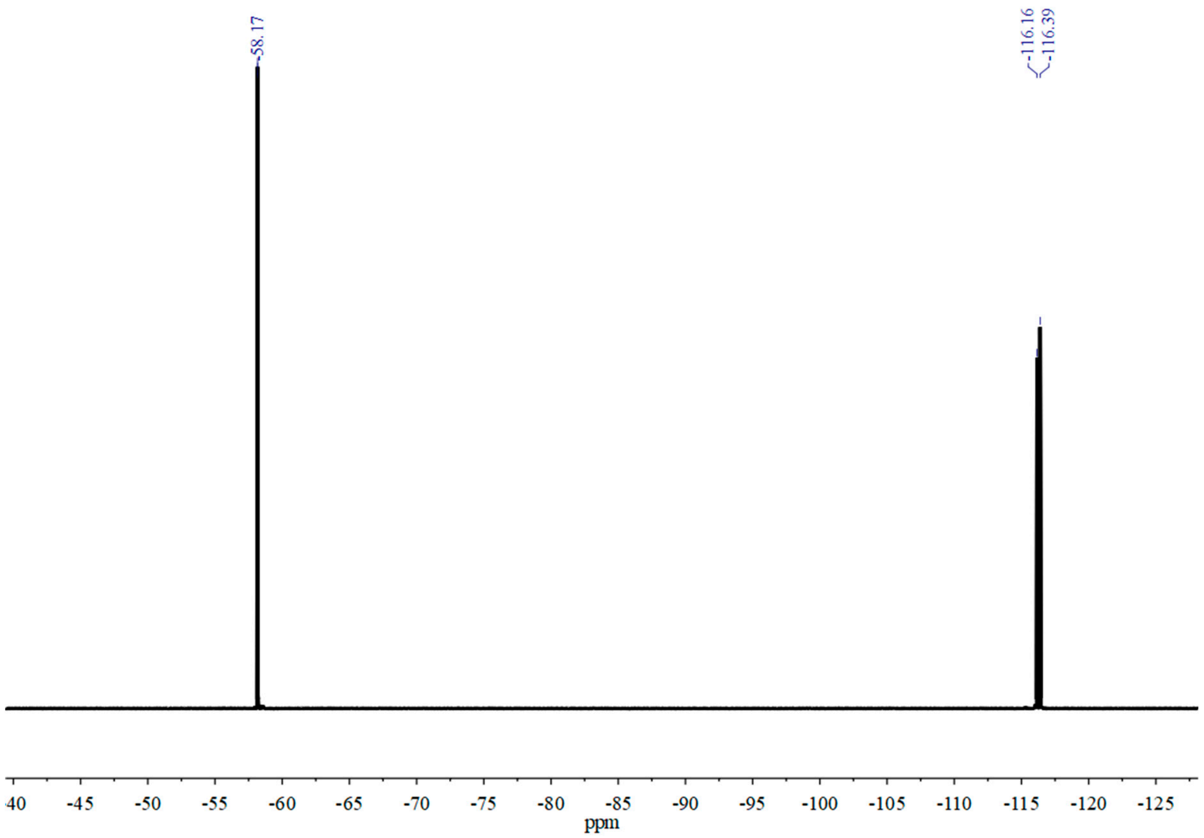

Figure S14 ^{19}F NMR spectrum of **L4** in $CDCl_3$ at room temperature.

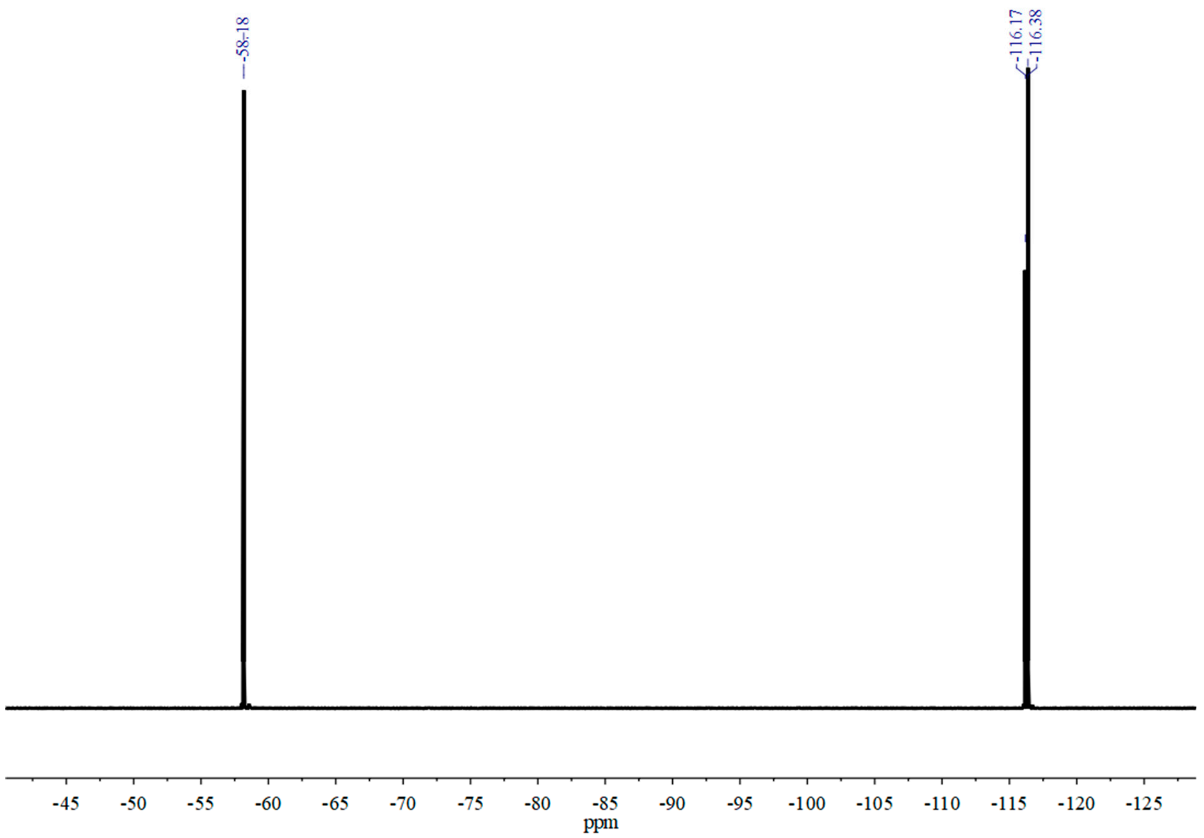

Figure S15 ^{19}F NMR spectrum of **L5** in $CDCl_3$ at room temperature.

5. $^{1}H/^{19}F$ NMR spectra of Ni1 – Ni5

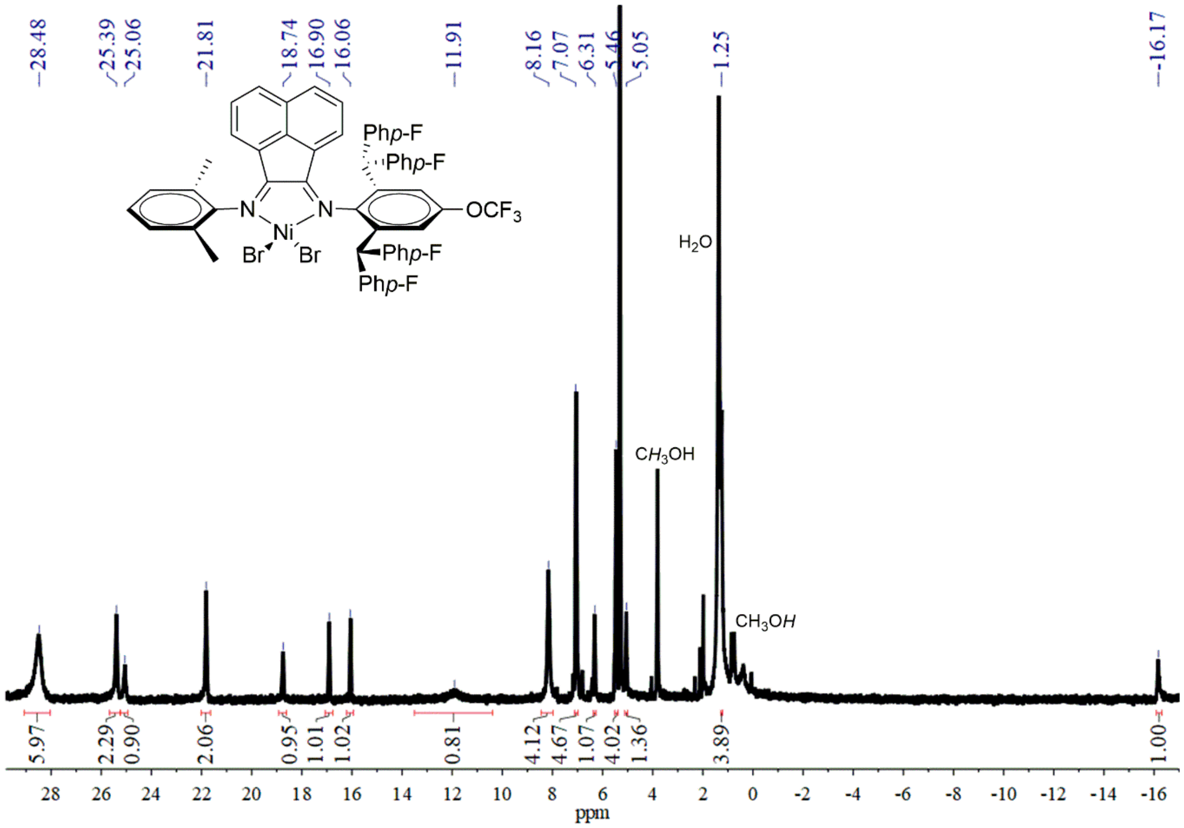

Figure S16 ^{1}H NMR spectrum of **Ni1** in CD_2Cl_2 at room temperature.

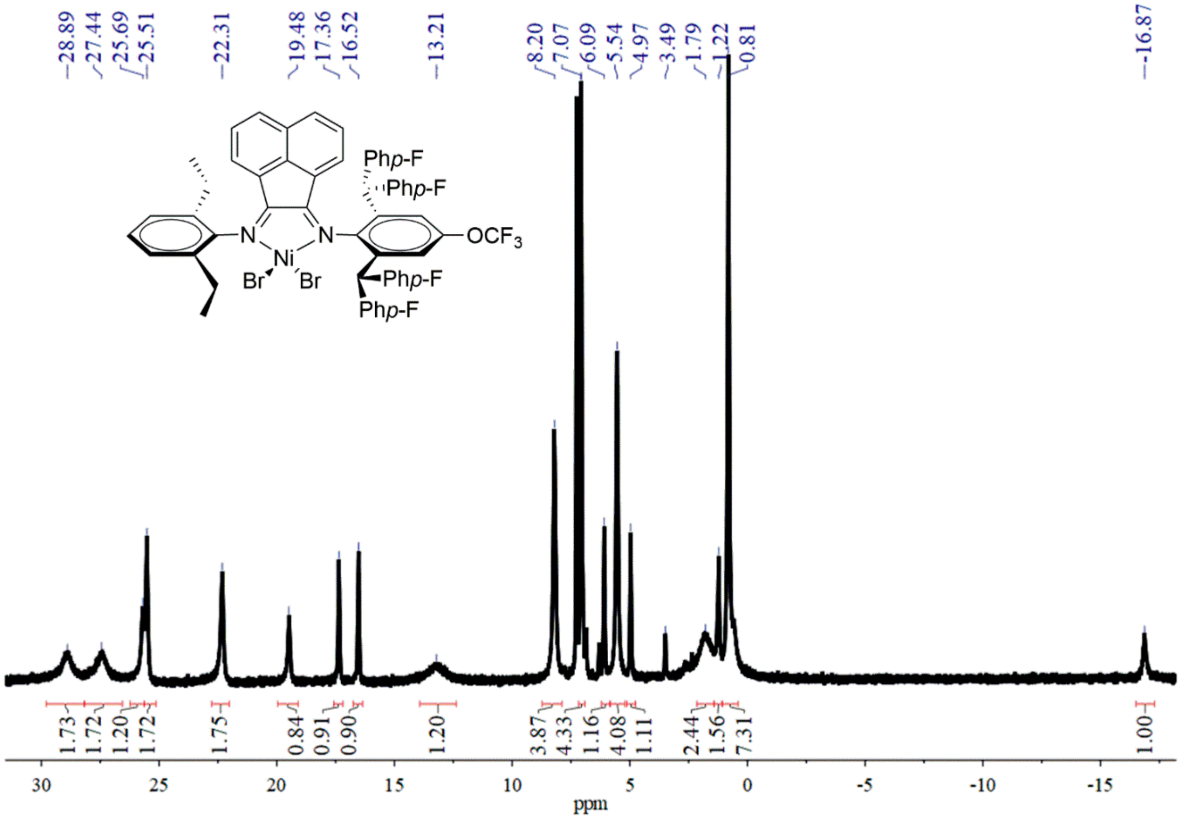

Figure S17 ^{1}H NMR spectrum of **Ni2** in $CDCl_3$ at room temperature.

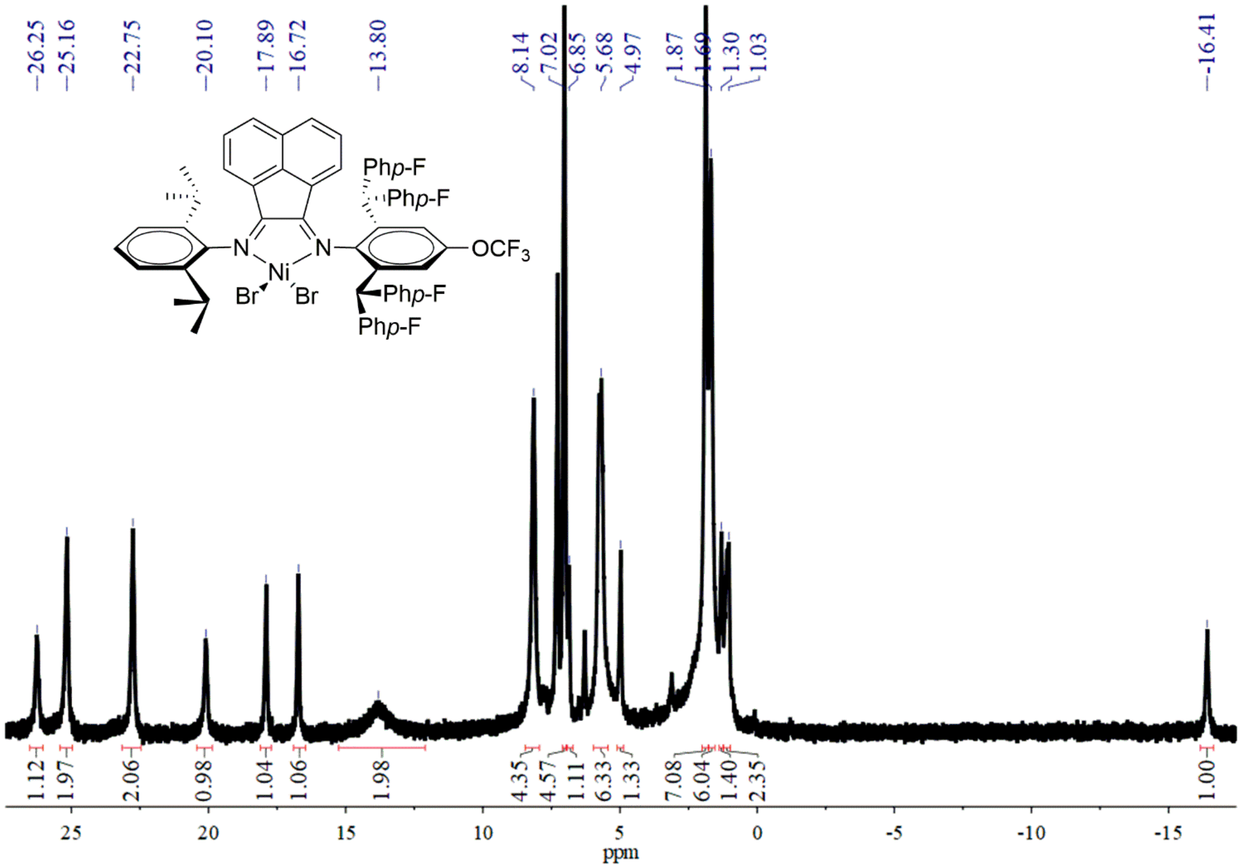

Figure S18 ^{1}H NMR spectrum of **Ni3** in $CDCl_3$ at room temperature.

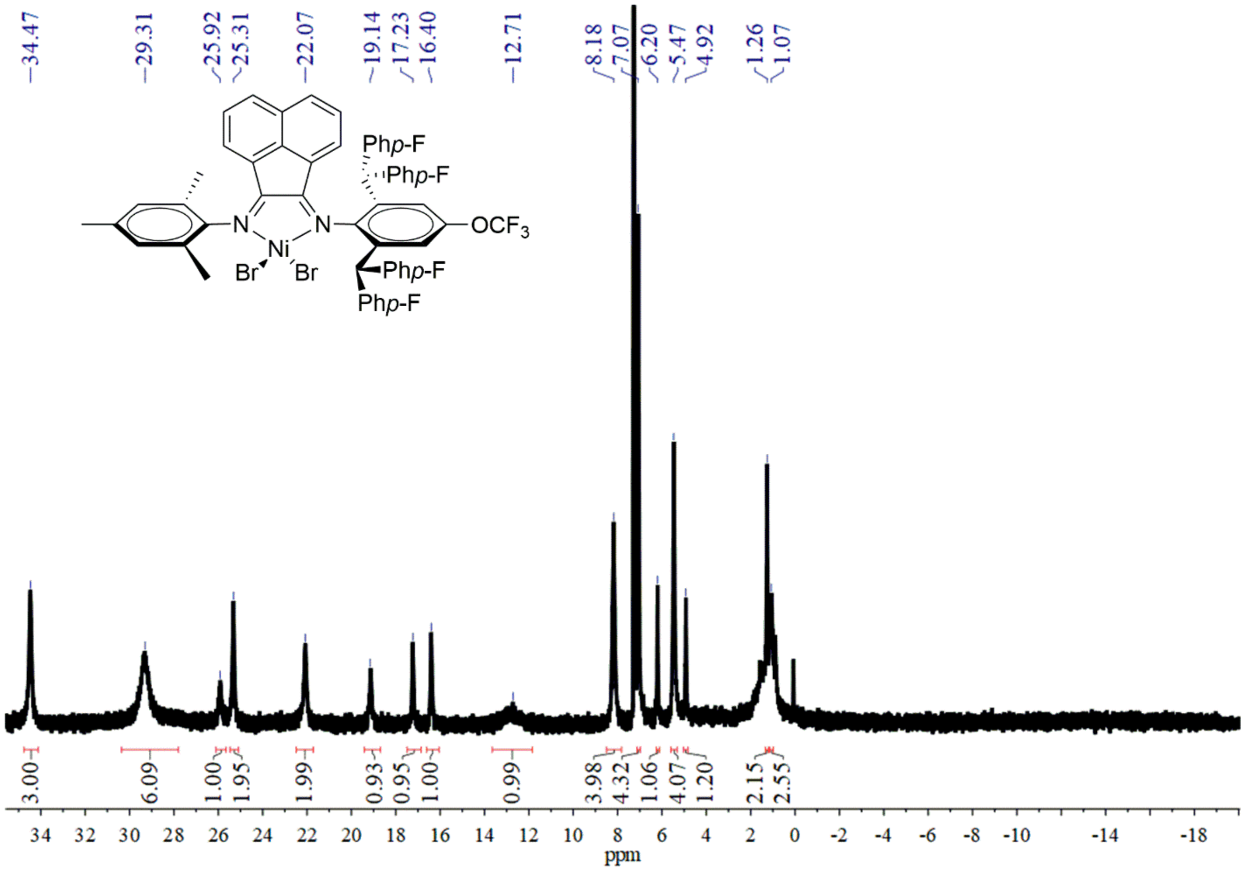

Figure S19 ^{1}H NMR spectrum of **Ni4** in $CDCl_3$ at room temperature.

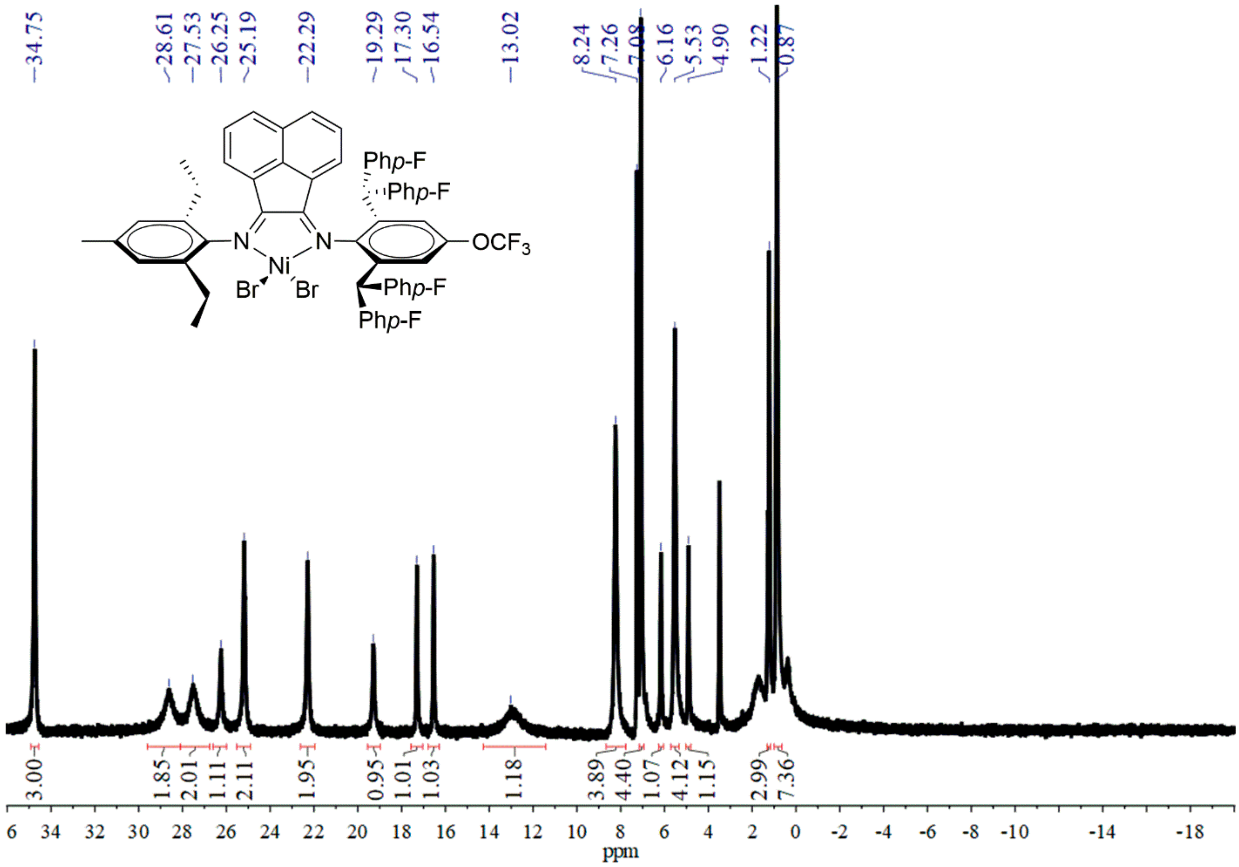

Figure S20 ^{1}H NMR spectrum of **Ni5** in $CDCl_3$ at room temperature.

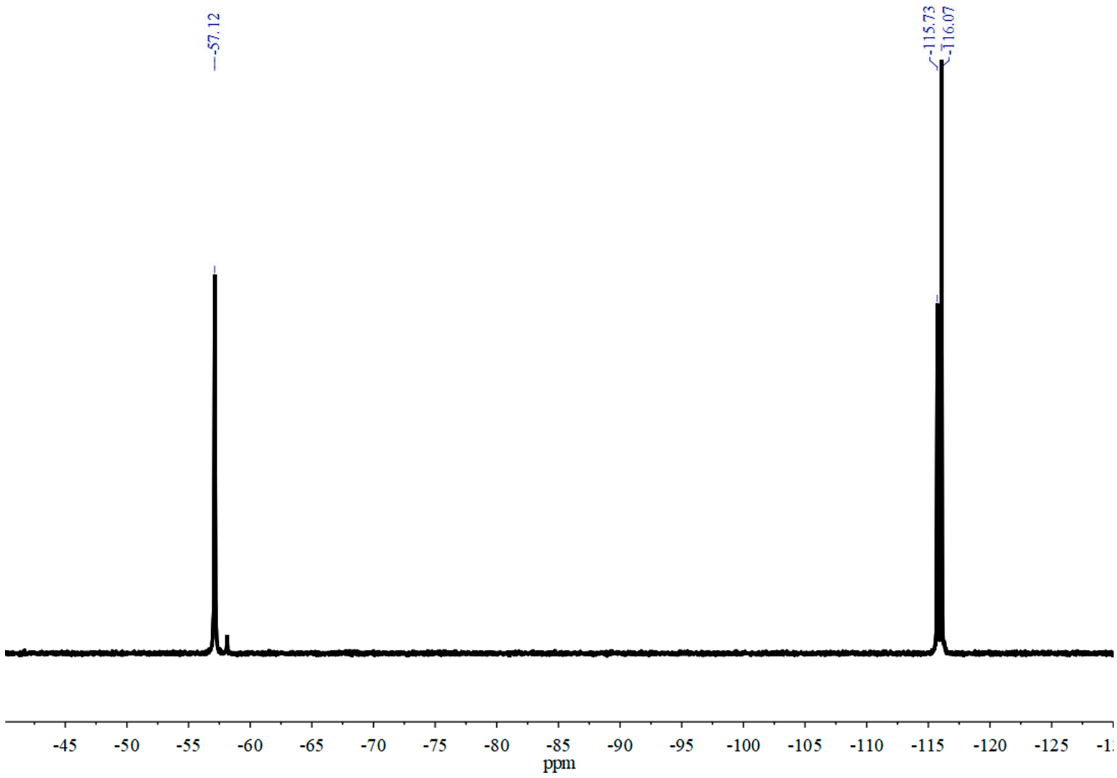

Figure S21 ^{19}F NMR spectrum of **Ni1** in $CDCl_3$ at room temperature.

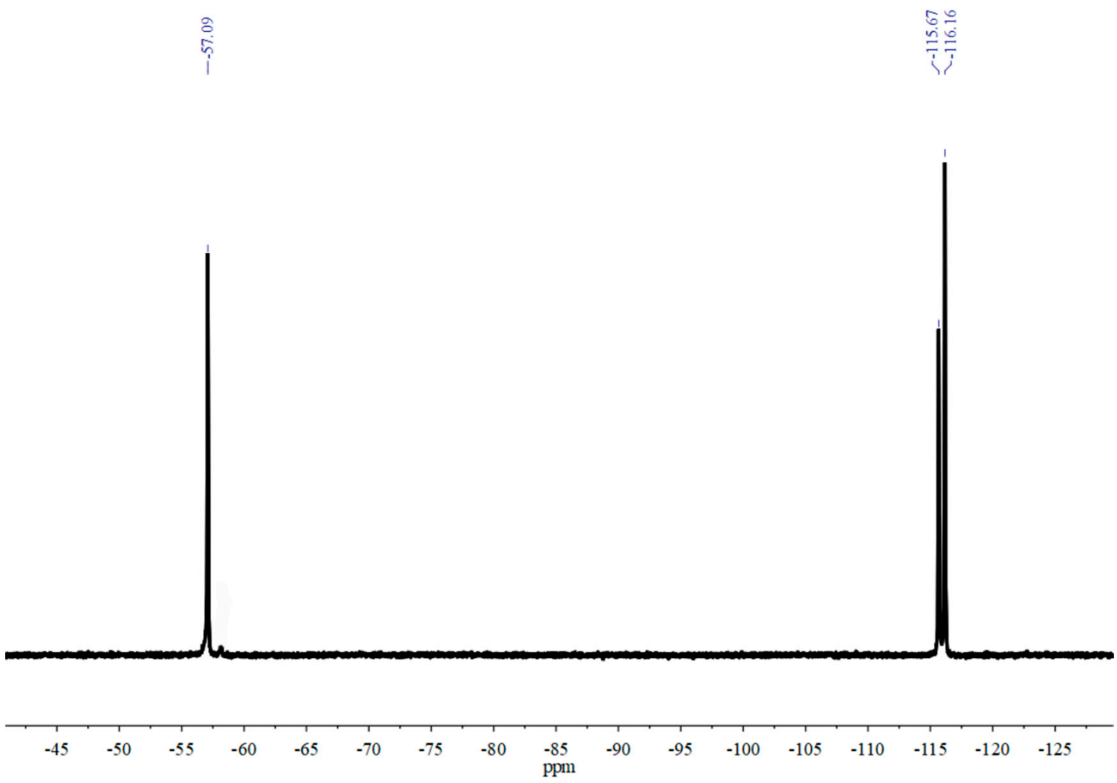

Figure S22 ^{19}F NMR spectrum of **Ni2** in $CDCl_3$ at room temperature.

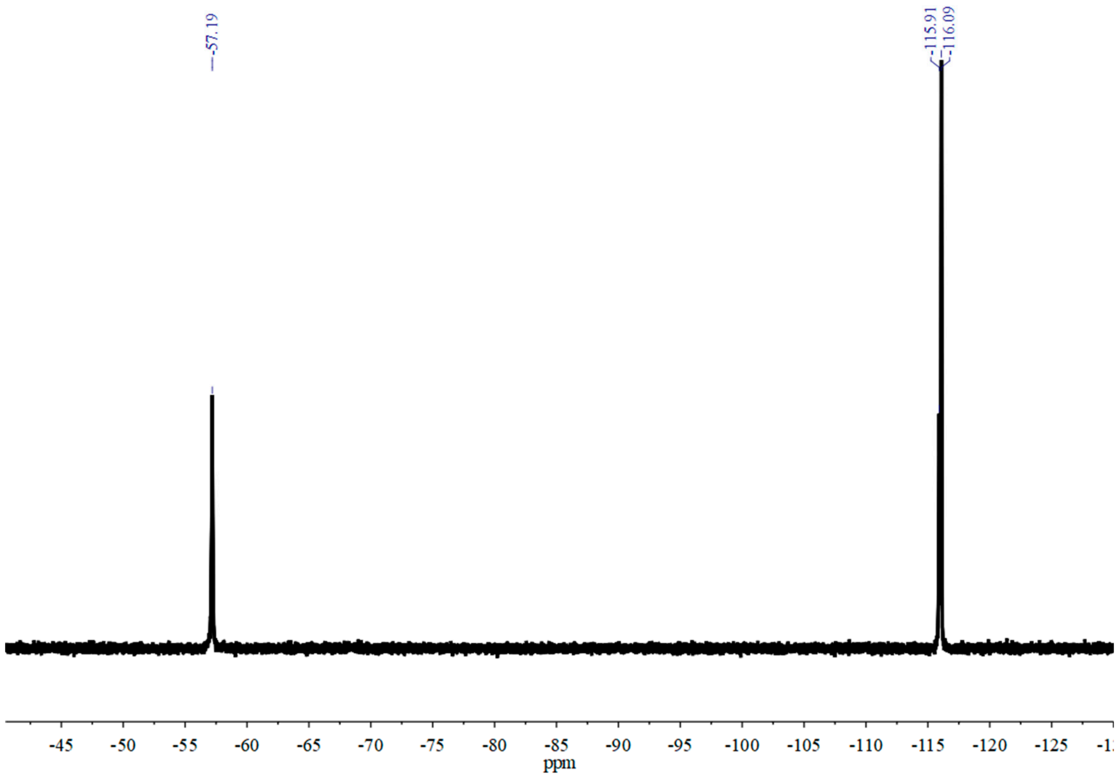

Figure S23 ^{19}F NMR spectrum of **Ni3** in $CDCl_3$ at room temperature.

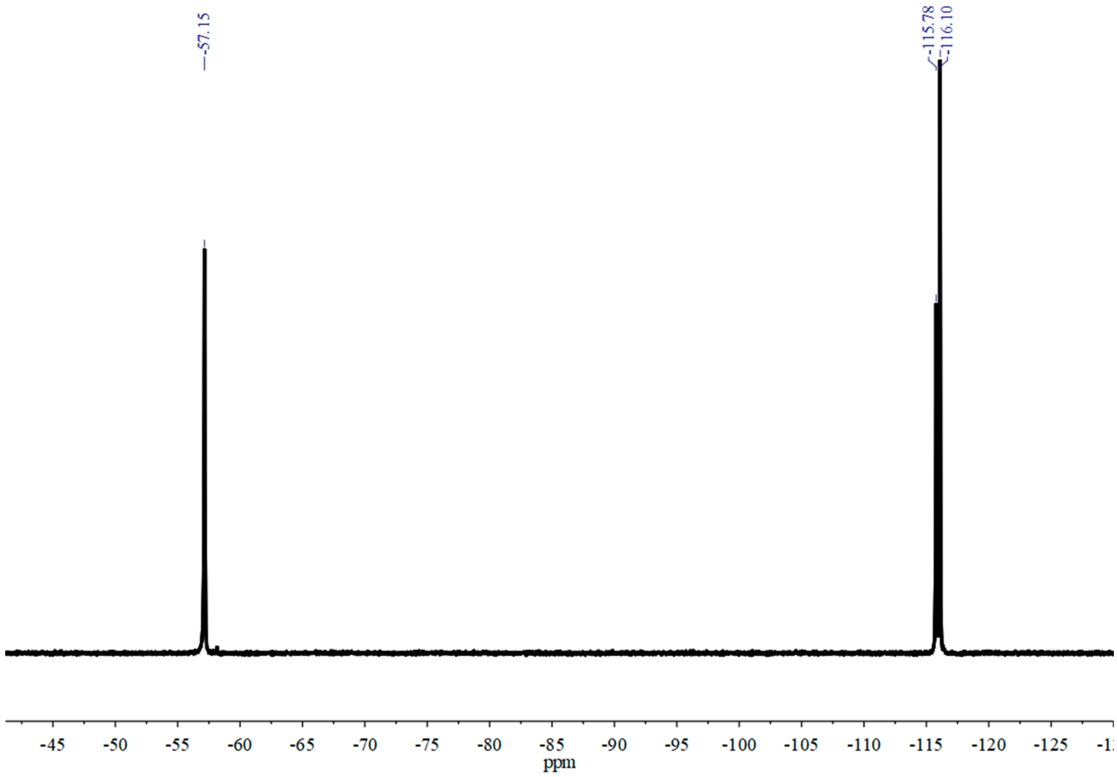

Figure S24 ^{19}F NMR spectrum of **Ni4** in $CDCl_3$ at room temperature.

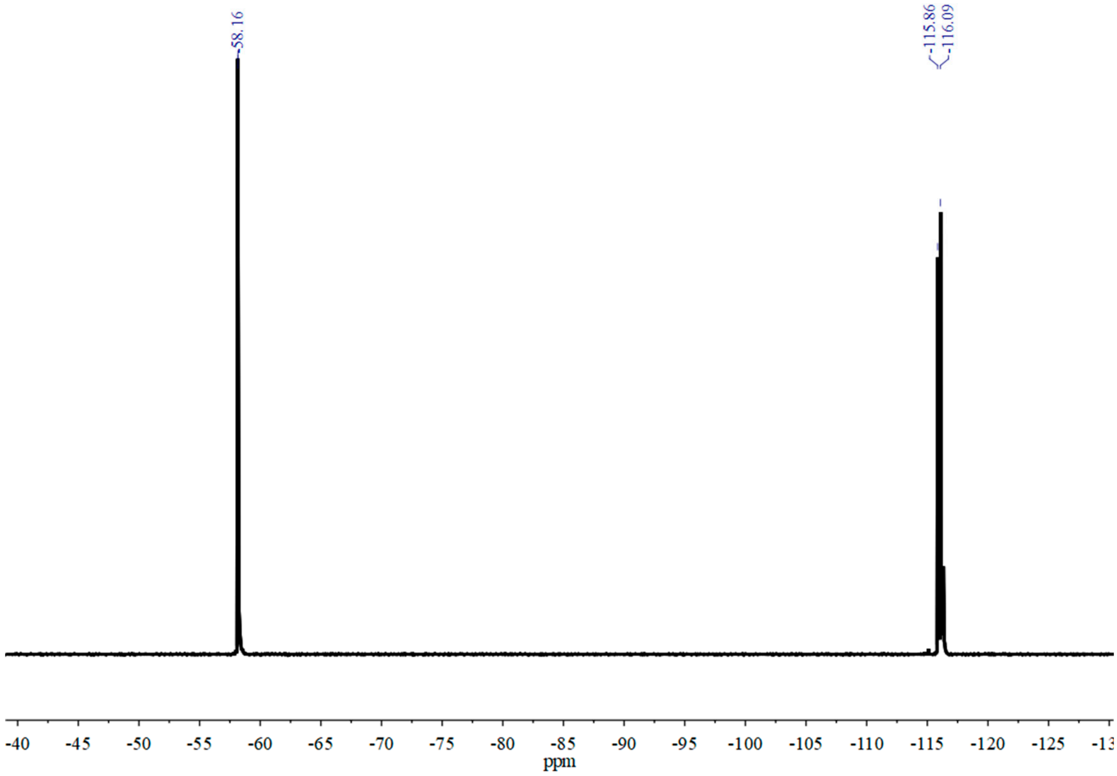

Figure S25 ^{19}F NMR spectrum of **Ni5** in $CDCl_3$ at room temperature.

6. GPC traces of the PE's obtained using Ni1/$EtAlCl_2$

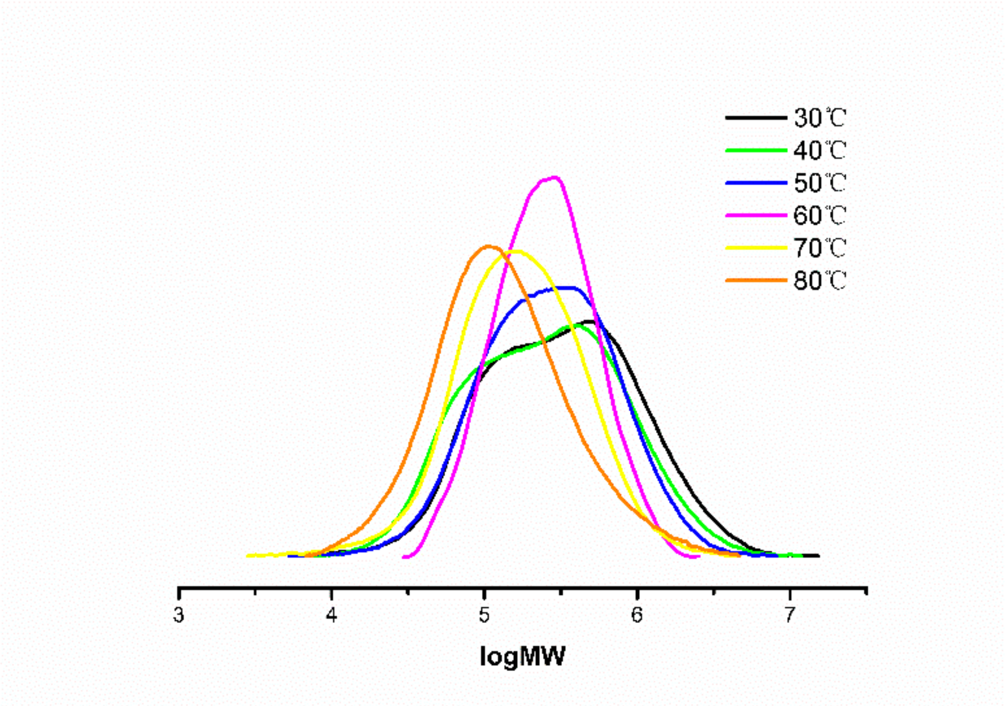

Figure S26 GPC traces of the PE's obtained using **Ni1**/$EtAlCl_2$ at different reaction temperatures (runs 1-6, Table 3).

7. ^{13}C NMR spectra of the polyethylenes

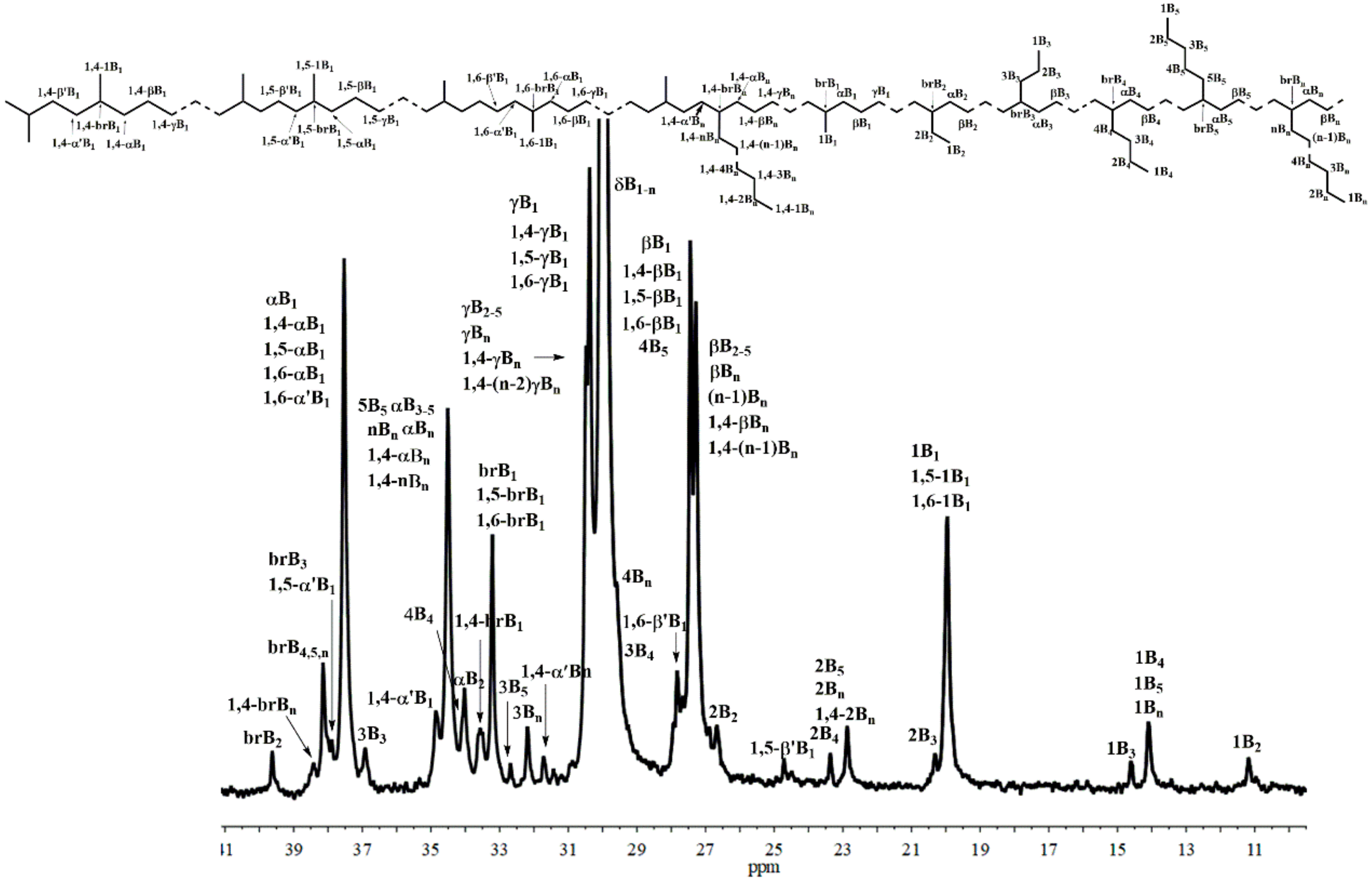

Figure S12 ^{13}C NMR spectrum of PE-60$_{EADC/300/T}$ along with a segment of the assigned polymer backbone (run 4, Table 3); recorded in $C_6D_4Cl_2$ at 100 °C.

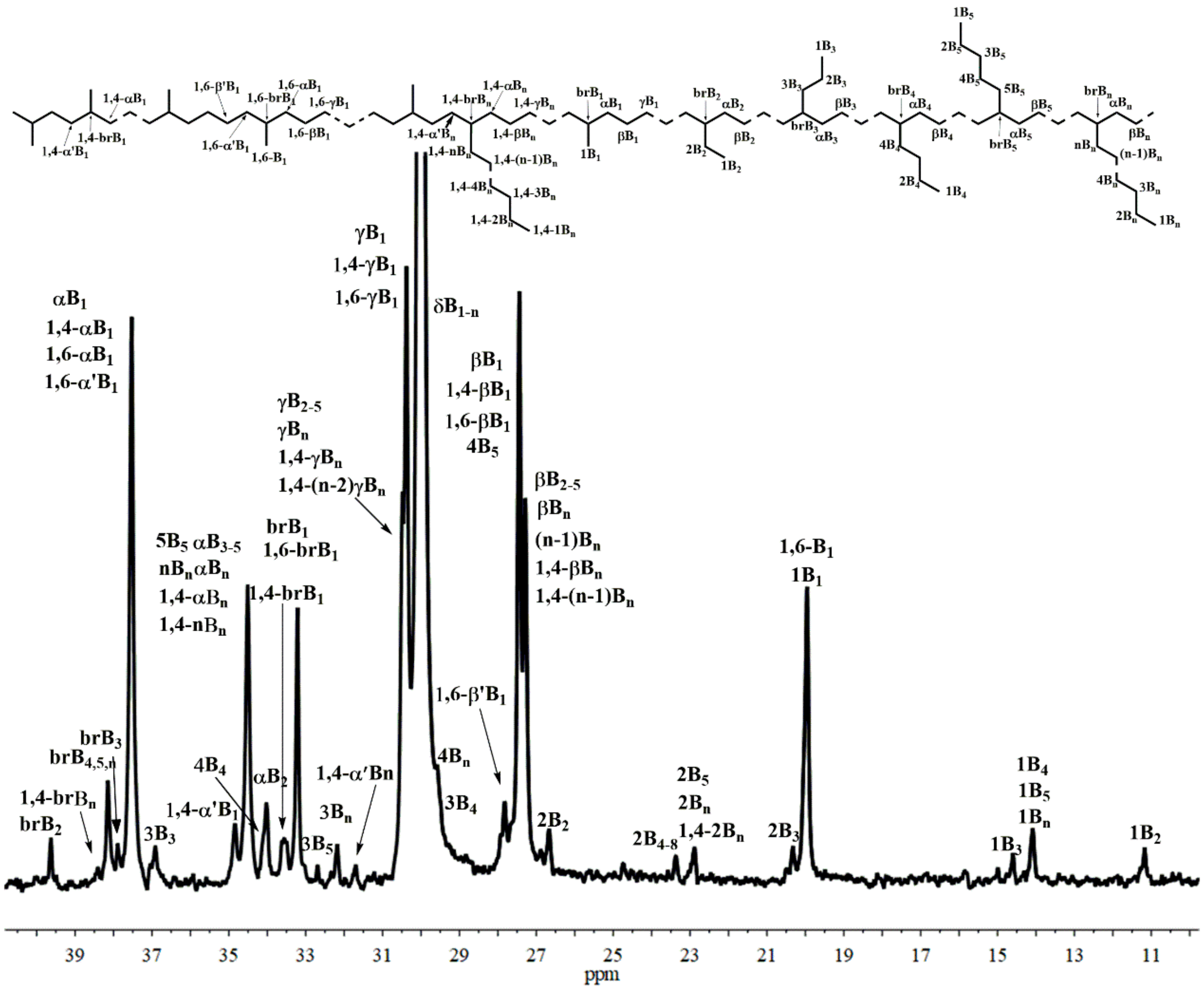

Figure S13 ^{13}C NMR spectrum of PE-40$_{EASC/300/T}$ along with a segment of the assigned polymer backbone (run 2, Table 4); recorded in $C_6D_4Cl_2$ at 100 °C.

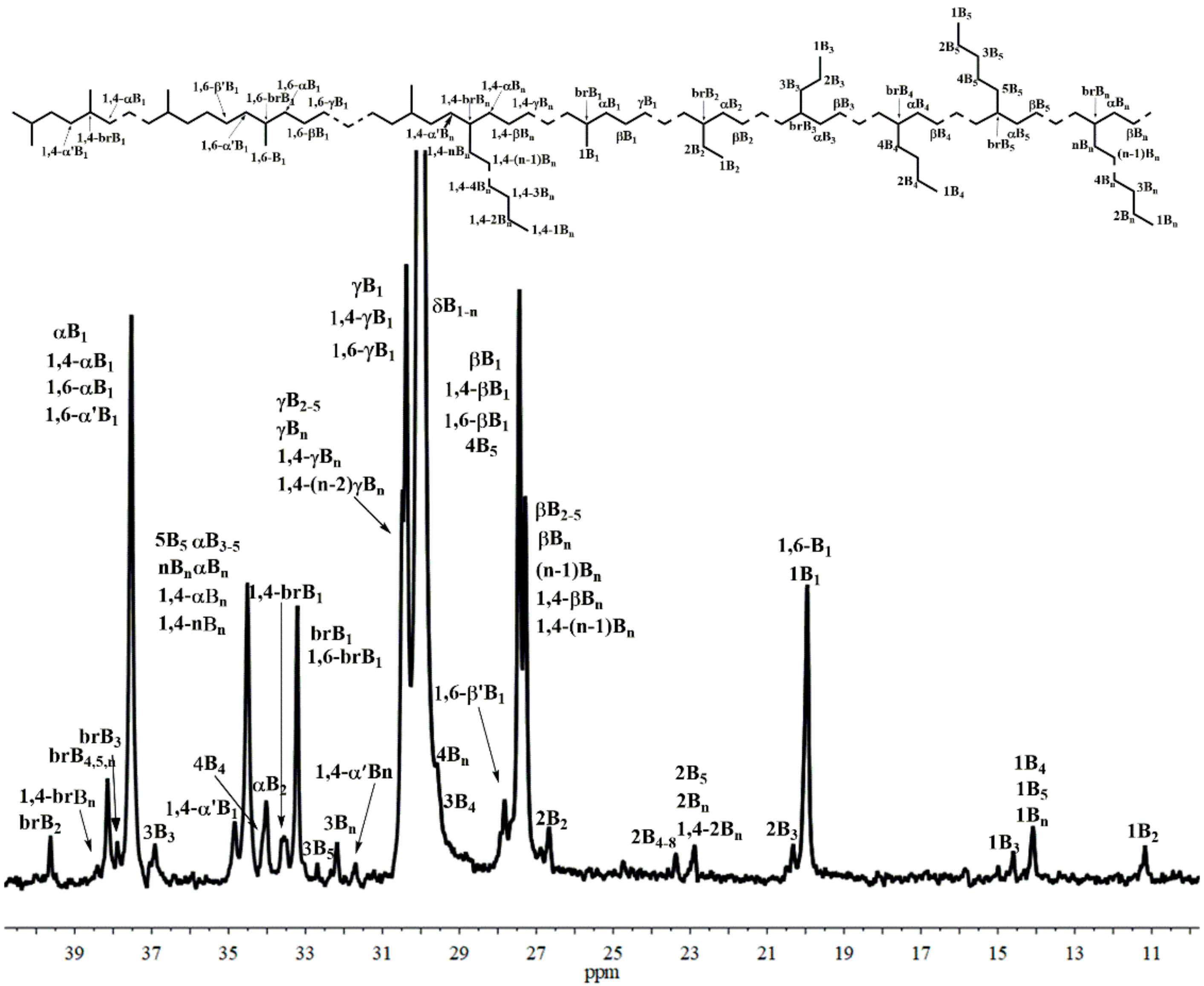

Figure S14 ^{13}C NMR spectrum of PE-60$_{EASC/300/T}$ along with a segment of the assigned polymer backbone (run 4, Table 4); recorded in $C_6D_4Cl_2$ at 100 °C.

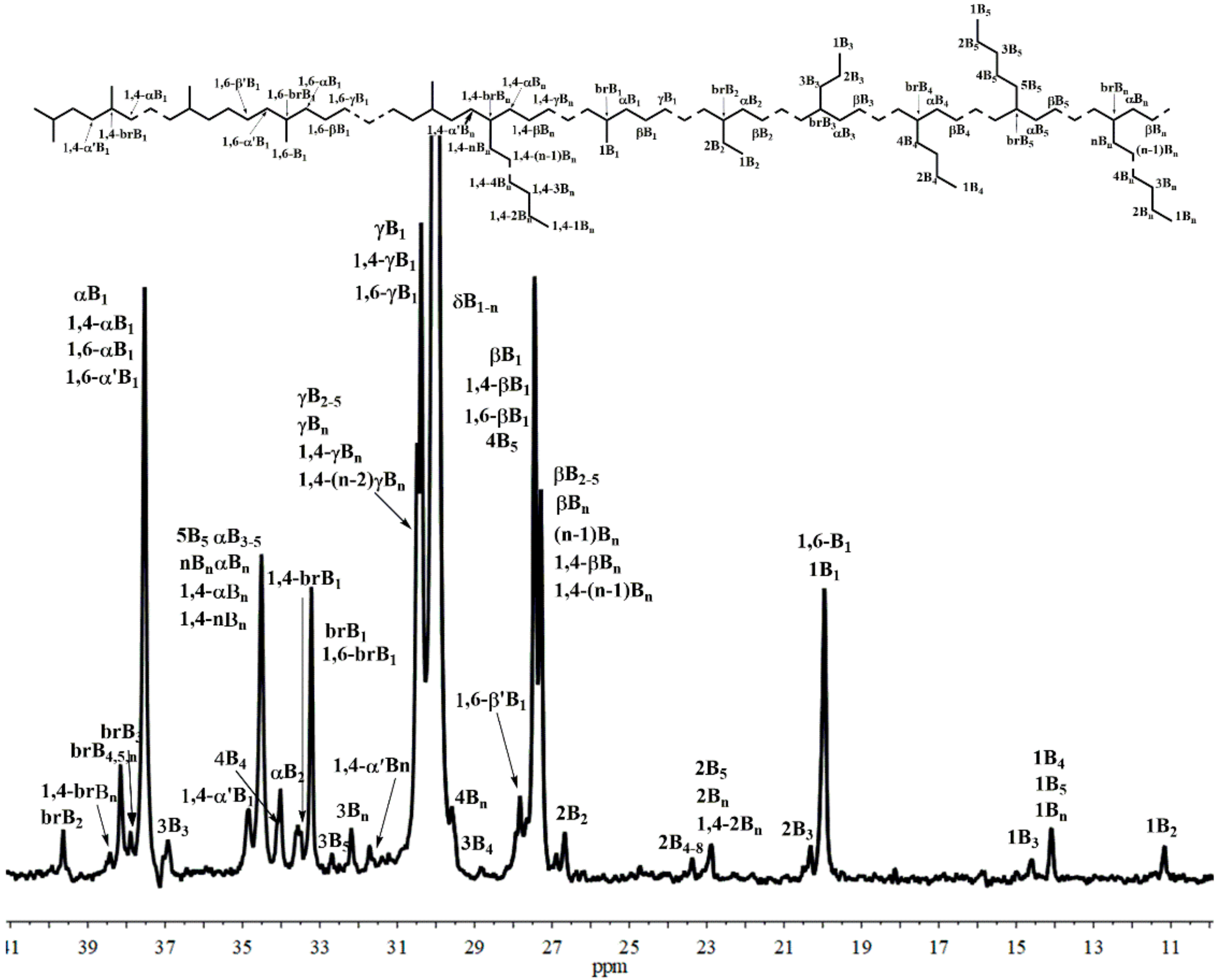

Figure S15 ^{13}C NMR spectrum of PE-40$_{EASC/450/T}$ along with a segment of the assigned polymer backbone (run 10, Table 4); recorded in $C_6D_4Cl_2$ at 100 °C.

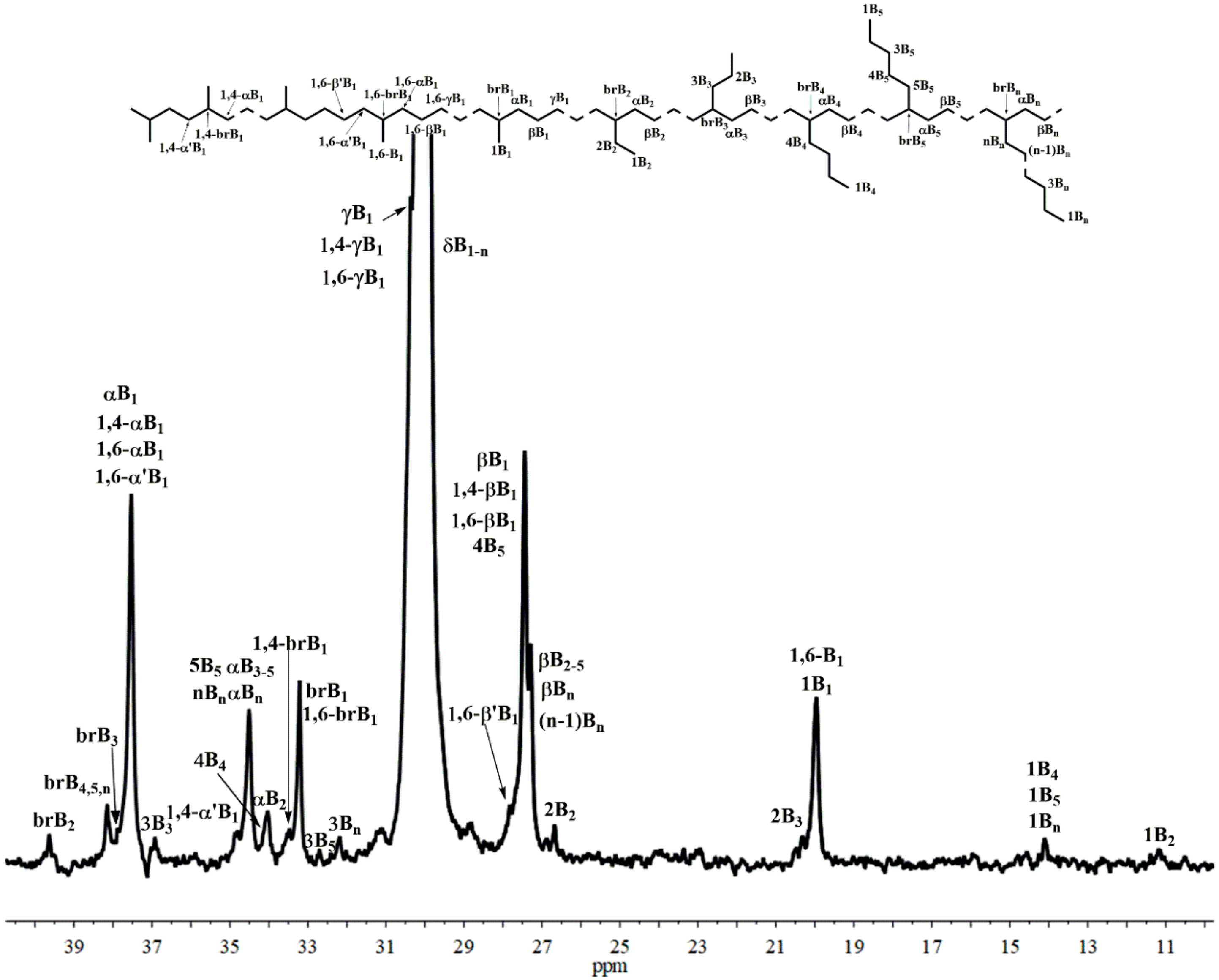

Figure S16 ^{13}C NMR spectrum of PE-40$_{EASC/450/H}$ along with a segment of the assigned polymer backbone (run 2, Table 5); recorded in $C_6D_4Cl_2$ at 100 °C.

8. References

1. Hosseinzadeh Z.; Liu M.; Zhang Q.; Liang T.; Solan G. A.; Ma Y.; Sun W.-H. Electronic Tuning of Sterically Encumbered 2-(Arylimino)Pyridine-Nickel Ethylene Polymerization Catalysts by Para-Group Modification. *Catalysts* **2022**, *12*, 1520.

2. Dolomanov O.V.; Bourhis L.J.; Gildea R.J.; Howard J.A.K.; Puschmann H. OLEX2: a complete structure solution, refinement and analysis program. *J. Appl. Cryst.* **2009**, *42*, 339-341.

3. Sheldrick G.M. SHELXT – Integrated space-group and crystalstructure determination. *Acta Cryst.* **2015**, *A71*, 3-8.

4. Sheldrick G.M. Crystal structure refinement with SHELXL. *Acta Cryst.* **2015**, *C71*, 3-8.
